# Clinical-genomic determinants of immune checkpoint blockade response in head and neck squamous cell carcinoma

**DOI:** 10.1172/JCI169823

**Published:** 2023-10-02

**Authors:** Cristina Valero, Mahdi Golkaram, Joris L. Vos, Bin Xu, Conall Fitzgerald, Mark Lee, Shannon Kaplan, Catherine Y. Han, Xin Pei, Reith Sarkar, Lillian A. Boe, Abhinav Pandey, Elizabeth S. Koh, Charlotte L. Zuur, David B. Solit, Traci Pawlowski, Li Liu, Alan L. Ho, Diego Chowell, Nadeem Riaz, Timothy A. Chan, Luc G.T. Morris

**Affiliations:** 1 Head and Neck Service, Immunogenomic Oncology Platform, Department of Surgery, Memorial Sloan Kettering Cancer Center (MSKCC), New York, New York, USA.; 2 Illumina Inc., San Diego, California, USA.; 3 Department of Pathology and Laboratory Medicine,; 4 Department of Radiation Oncology, and; 5 Department of Biostatistics and Epidemiology, MSKCC, New York, New York, USA.; 6 Department of Head and Neck Oncology and Surgery, Antoni van Leeuwenhoek Hospital–Netherlands Cancer Institute, Amsterdam, Netherlands.; 7 Department of Otorhinolaryngology and Head and Neck Surgery, Leiden University Medical Center, Leiden, Netherlands.; 8 Department of Medicine, MSKCC, New York, New York, USA.; 9 Department of Oncological Sciences, Icahn School of Medicine at Mount Sinai, New York, New York, USA.; 10 Lerner Research Institute, Cleveland Clinic, Cleveland, Ohio, USA.

**Keywords:** Immunology, Oncology, Cancer immunotherapy, Head and neck cancer

## Abstract

**BACKGROUND:**

Recurrent and/or metastatic (R/M) head and neck squamous cell carcinoma (HNSCC) is generally an incurable disease, with patients experiencing median survival of under 10 months and significant morbidity. While immune checkpoint blockade (ICB) drugs are effective in approximately 20% of patients, the remaining experience limited clinical benefit and are exposed to potential adverse effects and financial costs. Clinically approved biomarkers, such as tumor mutational burden (TMB), have a modest predictive value in HNSCC.

**METHODS:**

We analyzed clinical and genomic features, generated using whole-exome sequencing, in 133 ICB-treated patients with R/M HNSCC, of whom 69 had virus-associated and 64 had non-virus-associated tumors.

**RESULTS:**

Hierarchical clustering of genomic data revealed 6 molecular subtypes characterized by a wide range of objective response rates and survival after ICB therapy. The prognostic importance of these 6 subtypes was validated in an external cohort. A random forest-based predictive model, using several clinical and genomic features, predicted progression-free survival (PFS), overall survival (OS), and response with greater accuracy than did a model based on TMB alone. Recursive partitioning analysis identified 3 features (systemic inflammatory response index, TMB, and smoking signature) that classified patients into risk groups with accurate discrimination of PFS and OS.

**CONCLUSION:**

These findings shed light on the immunogenomic characteristics of HNSCC tumors that drive differential responses to ICB and identify a clinical-genomic classifier that outperformed the current clinically approved biomarker of TMB. This validated predictive tool may help with clinical risk stratification in patients with R/M HNSCC for whom ICB is being considered.

**FUNDING:**

Fundación Alfonso Martín Escudero, NIH R01 DE027738, US Department of Defense CA210784, The Geoffrey Beene Cancer Research Center, The MSKCC Population Science Research Program, the Jayme Flowers Fund, the Sebastian Nativo Fund, and the NIH/NCI Cancer Center Support Grant P30 CA008748.

## Introduction

Head and neck squamous cell carcinoma (HNSCC) describes a group of tumors that arise from the mucosal lining of the head and neck — specifically, the oral cavity, oropharynx, larynx, hypopharynx, nasopharynx, and sinonasal cavities. This cancer is the seventh most common cause of cancer death globally and is responsible for 4% of cancer deaths in the United States (1). Of the more than 400,000 people who will be diagnosed with HNSCC globally this year, more than half will experience a recurrence, usually within 2 years. Recurrent or metastatic (R/M) tumors are generally incurable, with a median survival of only 10 months (2–5). Immune checkpoint blockade (ICB) has improved outcomes for R/M HNSCC, but only 15% to 20% of patients respond; most patients do not experience a tumor response or any clinical benefit, while being at risk of developing immune-related adverse events (6, 7). Current immunotherapy strategies — broad application of ICB drugs in unselected patients — thus expose most patients to toxicity without benefit, at significant cost ($100,000 per quality-adjusted life year gained) (8).

Ideally, ICB drugs would be used with greater precision — earlier use in patients likely to benefit, with preferential use of other agents in patients for whom benefit is unlikely. However, there are no validated predictive biomarkers for ICB in HNSCC. The only FDA-approved clinical biomarker for ICB response in solid tumors is a high tumor mutational burden (TMB). Unfortunately, the predictive value of TMB in HNSCC is modest (9). Similarly, the predictive value of programmed death ligand 1 (PD-L1) expression on IHC analyses is low: in meta-analyses, PD-L1 underperforms compared with TMB (10). In HNSCC, the predictive value of PD-L1 is lower than for other cancer types (10) and is low overall (11, 12). Nonetheless, a PD-L1 combined positive score (CPS) of 1 or higher does correlate with clinical benefit to PD-1 inhibitor drugs and, while imperfect, is widely available to clinicians (13).

More in-depth characterization of clinical and genomic data from ICB-treated HNSCC patients is needed. To date, few studies have analyzed these features in patients with HNSCC (14–16). Ideal biomarkers suitable for clinical decision-making would use data potentially available in routine oncologic care: clinical characteristics, laboratory values, and DNA-derived genomic profiling.

To investigate the clinical and genomic biomarkers with the highest predictive value in HNSCC, we analyzed whole-exome sequencing (WES), clinical, and routine laboratory testing data from 133 patients with R/M HNSCC treated with ICB. These data were used to define molecular subtypes of HNSCC with relevance to ICB response, to construct a predictive model that was validated in an independent data set, and, finally, to generate a well-curated clinical-genomic data resource useful for further biomarker development and for guiding the next steps of therapeutic investigation of R/M HNSCC.

## Results

### Characteristics and outcomes of an ICB-treated cohort of patients with HNSCC.

The clinical characteristics of 133 patients with R/M HNSCC treated with ICB are shown in Table 1. Most patients received anti–programmed cell death 1 (anti–PD-1) or anti–PD-L1 monotherapy (126 of 133, 95%), 1 patient (1%) received anticytotoxic T lymphocyte–associated protein 4 (CTLA-4) monotherapy, and 6 patients (5%) were treated with concurrent anti–PD-1/anti–PD-L1 and anti–CTLA-4. Most patients (129, 97%) had previously received chemotherapy. Of these, 122 patients (95%) received platinum-based chemotherapy, with 48 patients (39%) starting immunotherapy within 6 months of the last platinum dose.

**Figure 1:**
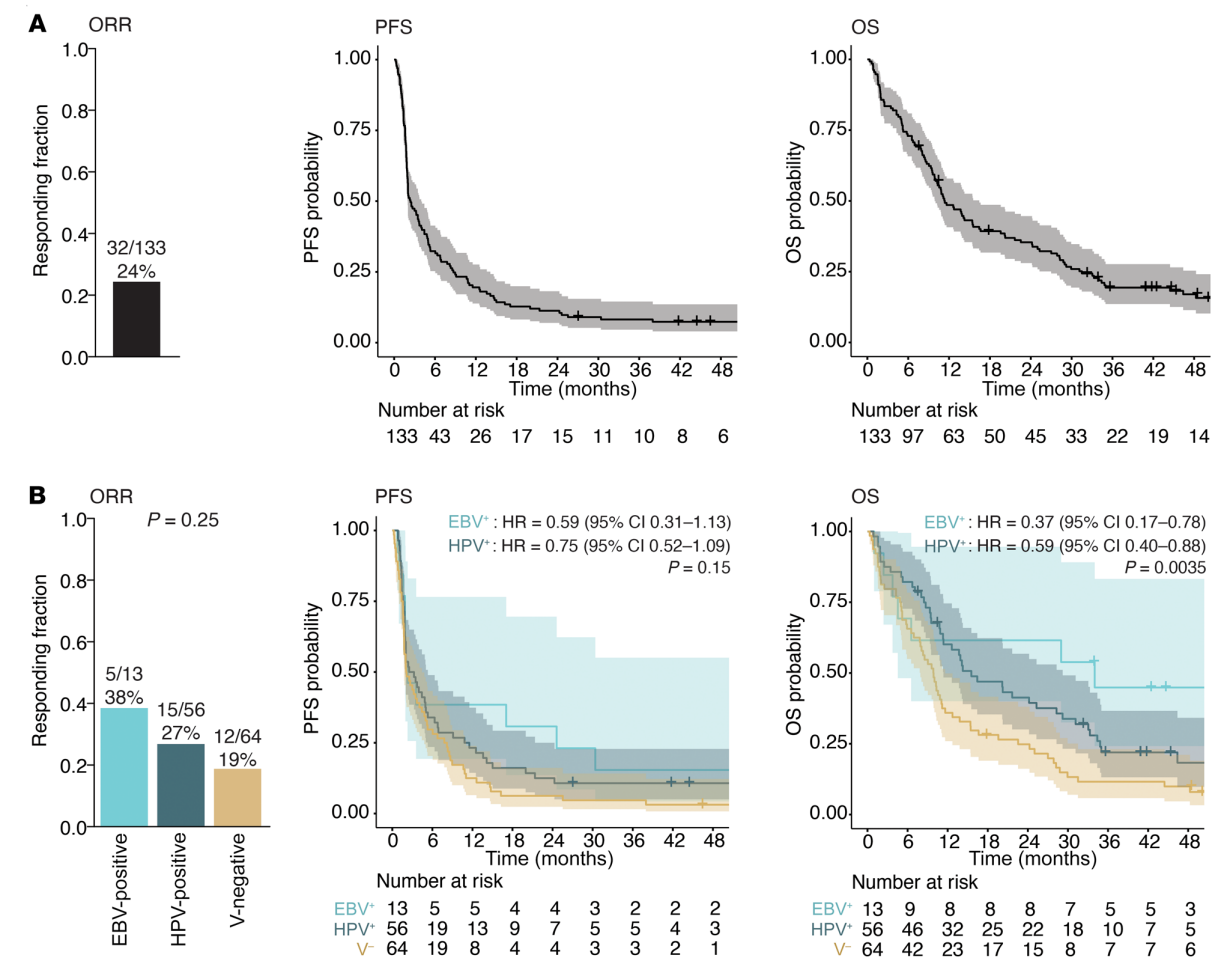
Response and survival outcomes for 133 patients with R/M HNSCC (64 V-negative, 56 HPV-positive, and 13 EBV-positive) treated with ICB. (**A**) ORR and Kaplan-Meier estimates for PFS and OS following ICB treatment for all R/M HNSCC patients. (**B**) ORR, PFS, and OS data for V-negative, HPV-positive, and EBV-positive tumors separately. The *P* value in the ORR bar chart was obtained with a Fisher’s exact test with Freeman-Halton extension. HRs and 95% CIs for HPV-positive and EBV-positive tumors were calculated relative to V-negative tumors using Cox regression analysis. *P* values in PFS and OS Kaplan-Meier plot were calculated using a log-rank test.

The objective response rate (ORR, defined as a complete or partial response by Response Evaluation Criteria in Solid Tumors 1.1 [RECIST 1.1] criteria) (17) was 24% (32 patients), and the rate of clinical benefit (defined as an objective response or stable disease >6 months) was 28% (37 patients). Kaplan-Meier estimates of the 6-month, 1-year, and 2-year progression-free survival (PFS) and overall survival (OS) are shown in Figure 1A and Table 2.

A subgroup of HNSCC tumors develop from infection with oncogenic viruses — HPV (most commonly in oropharyngeal tumors) or EBV (in nasopharyngeal carcinoma). Virus-associated tumors have prognostic profiles distinct from those of tumors with a nonviral etiology (2, 18, 19). In this study, 64 tumors (48%) were not associated with oncogenic viruses (virus-negative [V-negative]), 56 tumors (42%) were HPV associated (48 oropharynx, 8 from other subsites), and 13 tumors (10%) were EBV associated (all nasopharynx). The ORR was numerically higher in patients with EBV-positive (38%) or HPV-positive (27%) tumors compared with virus-negative tumors (19%), but this difference was not statistically significant (*P* = 0.25) (Figure 1B). Similarly, PFS did not differ to a statistically significant degree based on viral status. However, OS was significantly superior in both the EBV-positive (HR, 0.37 [95% CI: 0.17–0.78]) and HPV-positive (HR, 0.59 [95% CI: 0.40–0.88]) populations compared with the V-negative population (Figure 1B). PFS and OS were not significantly different between the EBV-positive (median, 2.3 [95% CI: 1.9–∞] and 34.0 [95% CI: 4.5–∞] months, respectively) and HPV-positive populations (median, 2.9 [95% CI: 1.9–5.3] and 15.5 [95% CI: 11.4–29.2] months, respectively). Therefore, based on the similar response, progression, and survival outcomes, EBV-positive and HPV-positive subpopulations were categorized together as virus-associated (virus-positive [V-positive]) HNSCC for downstream analyses (Table 2 and Supplemental Figure 1A; supplemental material available online with this article; https://doi.org/10.1172/JCI169823DS1).

A prior study of ICB-treated melanoma patients demonstrated an association between the occurrence of immune-related adverse events (irAEs) and recurrence-free survival (20). In this R/M HNSCC cohort, 23% (30 of 133) of patients experienced irAEs of any grade after ICB treatment. These patients had a significantly higher ORR than did those without irAEs (50% vs. 17%; *P* = 4.3 × 10^–4^). Similarly, 1-year PFS (30 vs. 17%; *P* = 0.005) and OS (67 vs. 43%; *P* = 0.044) were significantly superior in the subgroup experiencing irAEs. However, these differences were not observed after correcting for possible immortal time bias by modeling toxicity as a time-dependent covariate (HR, 1.26 [95% CI: 0.74–2.12]; *P* = 0.40 for PFS; HR, 0.91 [95% CI: 0.56–1.48]; *P* = 0.71 for OS).

### Differences in the genomic landscape based on viral etiology.

**Figure 2:**
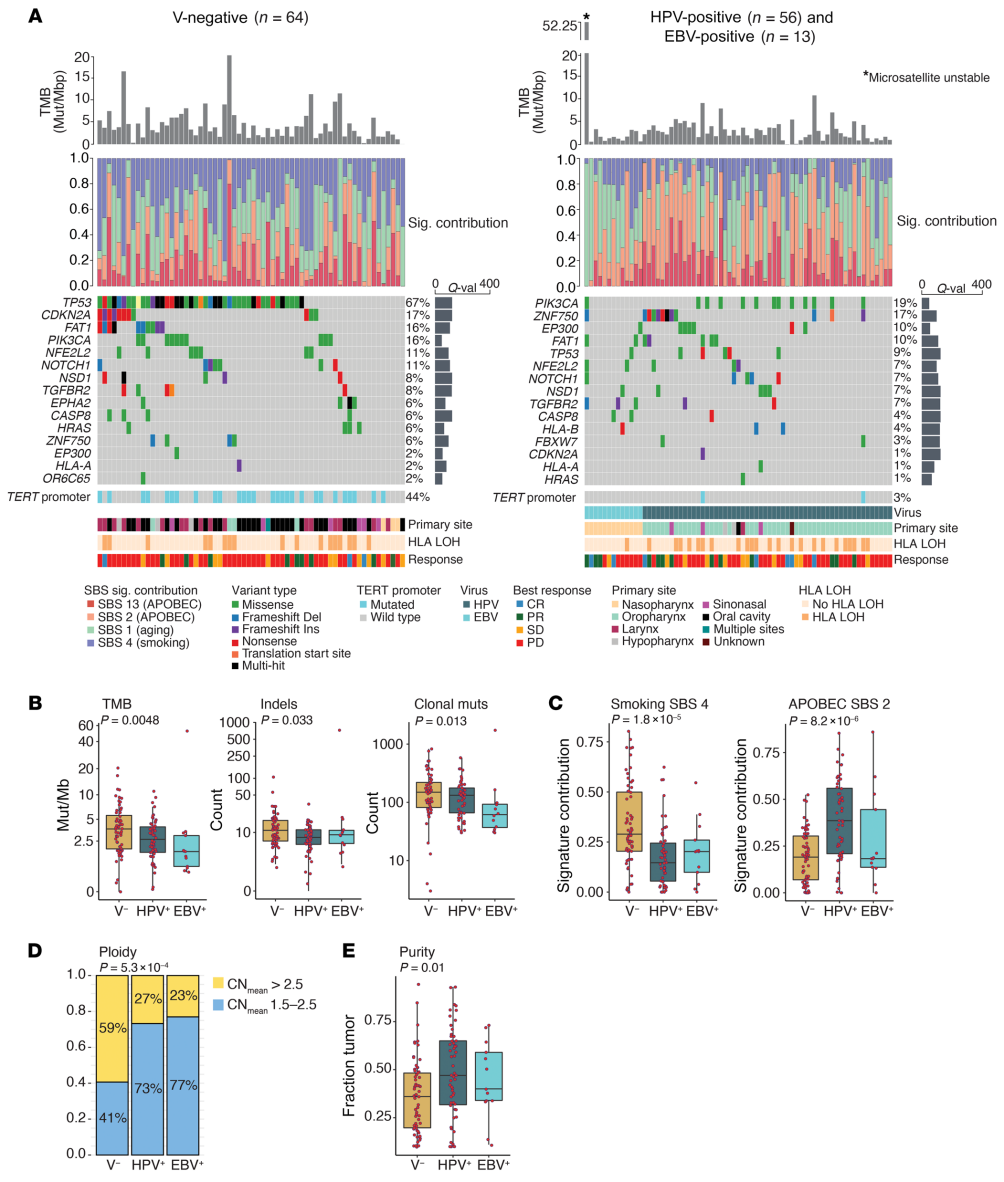
The genomic landscape of V-negative, HPV-positive, and EBV-positive HNSCC. (**A**) Each column represents a unique sample. V-negative tumors are shown on the left and HPV-positive and EBV-positive tumors on the right. The top bar chart represents the TMB in mutations/Mbp per sample; the EBV-positive sample marked with an asterisk was microsatellite unstable. Below that, a stacked bar chart shows the proportion of the total mutational load attributed to COSMIC signatures (23) associated with APOBEC activity, smoking, or aging. Oncoprints show the top 15 most frequently mutated genes (listed on the left), the variant type (box color), the total proportion of samples with a mutation in that gene (percentage on the right), and the *Q* value per gene (bar chart on the right). Tracks below the oncoprints show mutations in the *TERT* promoter region; an individual tumor’s causative virus (in V-positive tumors only); the primary tumor site; the proportion of LOH at the HLA locus (51); and the tumors’ best objective response. Ins, insertion; Del, deletion; Sig., signature; val, value.(**B**) Box plots show (from left to right) the TMB, the sum of insertions and deletions per exome, and the total number of clonal mutations per exome in V-negative, HPV-positive, and EBV-positive tumors. The clonal mutational load was available for 124 samples. *P* values were calculated using a Kruskal-Wallis test. (**C**) Box plots show the contribution of an SBS signature associated with smoking (SBS 4) and APOBEC activity (SBS 2) in V-negative, HPV-positive, and EBV-positive tumors (*n* = 133). *P* values were calculated using a Kruskal-Wallis test. (**D**) Stacked bar chart shows the proportion of diploidy (blue, mean copy number [CN] of 1.5–2.5) and hyperploidy (yellow, mean CN >2.5) in V-negative, HPV-positive, and EBV-positive tumors (*n* = 133). The *P* value was calculated using a Fisher’s exact test with Freeman-Halton extension. (**E**) Box plots show the sample tumor purity estimates derived from FACETS in V-negative, HPV-positive, and EBV-positive tumors (*n* = 133). The *P* value was calculated using a Kruskal-Wallis test.

In 64 V-negative, 56 HPV-positive, and 13 EBV-positive tumors, DNA from biopsies was analyzed using deep WES (Figure 2A and Supplemental Figure 1B), with median coverage 776× (IQR: 586–867×). While 1 EBV-positive HNSCC sample was microsatellite unstable (Figure 2A) and had an exceptionally high TMB (52.25 mutations/megabase pair [muts/Mbp]), the cohort median TMB was low (3.04 muts/Mbp). In keeping with previous reports (16, 21, 22), we found that the median TMB differed significantly by viral status: highest in V-negative HNSCC (3.72 muts/Mbp [IQR: 1.88–5.60]), followed by HPV-positive (2.66 mut/Mb [IQR: 1.65–3.97]), and EBV-positive tumors (1.70 mut/Mb [IQR: 0.87–3.01]; *P* = 0.0048). We observed similar differences for median numbers of indels and clonal mutations per sample (Figure 2B). TMB varied significantly across HNSCCs from different primary sites (*P* = 7.8 × 10^–5^) (Supplemental Figure 1C) and was higher in patients with a 10-or-more pack-year smoking history (median, 3.6 vs. 2.2; *P* = 0.014), among which laryngeal tumors were significantly overrepresented (26% vs. 4%; *P* < 0.001).

When considering mutated genes individually, V-negative HNSCCs were characterized by prevalent (67%) *TP53* mutations and frequent mutations in *CDKN2A* (17%) (Figure 2A). In HPV-positive HNSCC tumors, we observed missense mutations in *PIK3CA* in 21%, along with variants in *ZNF750* and *EP300*, and, in 20% and 11% of HNSCCs, respectively. The most frequently mutated gene in EBV-positive tumors was *TGFBR2* (23%). *TERT* promoter mutations were absent in EBV-positive samples and infrequent in HPV-positive samples (4%), but prevalent in V-negative HNSCCs (44%) (Figure 2A). *TERT* promoter mutations were mainly enriched in V-negative tumors of the oral cavity: 77% compared with 15% for V-negative HNSCCs in other sites (*P* = 7.3 × 10^–7^).

We identified the top 4 single base substitution (SBS) mutational signatures defined by the Catalogue of Somatic Mutations in Cancer (COSMIC) (23): signatures 1, 2, 4, and 13 (Figure 2A). A smoking signature (SBS 4) was more prevalent in V-negative than in HPV-positive or EBV-positive tumors (*P* = 1.8 × 10^–5^) (Figure 2C). Conversely, the contribution of signatures associated with the apolipoprotein B mRNA–editing enzyme catalytic polypeptide-like (APOBEC) (SBS 2 and SBS 13) was relatively low in V-negative and EBV-positive tumors and high in HPV-positive HNSCC (*P* = 8.2 × 10^–6^) (Figure 2C), as previously reported (24, 25).

The copy number profiles of V-negative, HPV-positive, and EBV-positive HNSCCs are shown in Supplemental Figure 1D. V-negative HNSCCs more frequently had a hyperploid (>2.5) (26) mean copy number (59%) compared with HPV-positive or EBV-positive tumors (27% and 23%, respectively; *P* = 5.3 × 10^–4^) (Figure 2D). Tumor purity was highest in HPV-positive tumors, followed by EBV-positive and V-negative tumors (*P* = 0.01) (Figure 2E). Deletion of 9p24.1 — the locus of *CD274* (PD-L1), *PDCD1LG2* (PD-L2), and *JAK2* — was observed in 23 (36%) of V-negative, 5 (9%) of HPV-positive, and 2 (15%) of EBV-positive tumors, a statistically significant difference (*P* = 0.0012). Of note, the fraction of samples with a copy number loss at the 9p24.1 locus was numerically higher in ICB nonresponders than responders with V-negative tumors (40% vs. 17%; *P* = 0.18) and EBV-positive tumors (25% vs. 0; *P* = 0.49), but not HPV-positive tumors (7% vs. 13%; *P* = 0.60) (Supplemental Figure 1E). The frequency of loss of heterozygosity (LOH) at 1 or more HLA class I loci, a cancer immune escape mechanism (27), significantly varied across HNSCCs, with the highest prevalence in HPV-positive tumors (41%), followed by V-negative (27%), and EBV-positive samples (8%; *P* = 0.038) (Figure 2A).

### Identifying molecular subtypes of HNSCC with relevance to ICB response.

**Figure 3:**
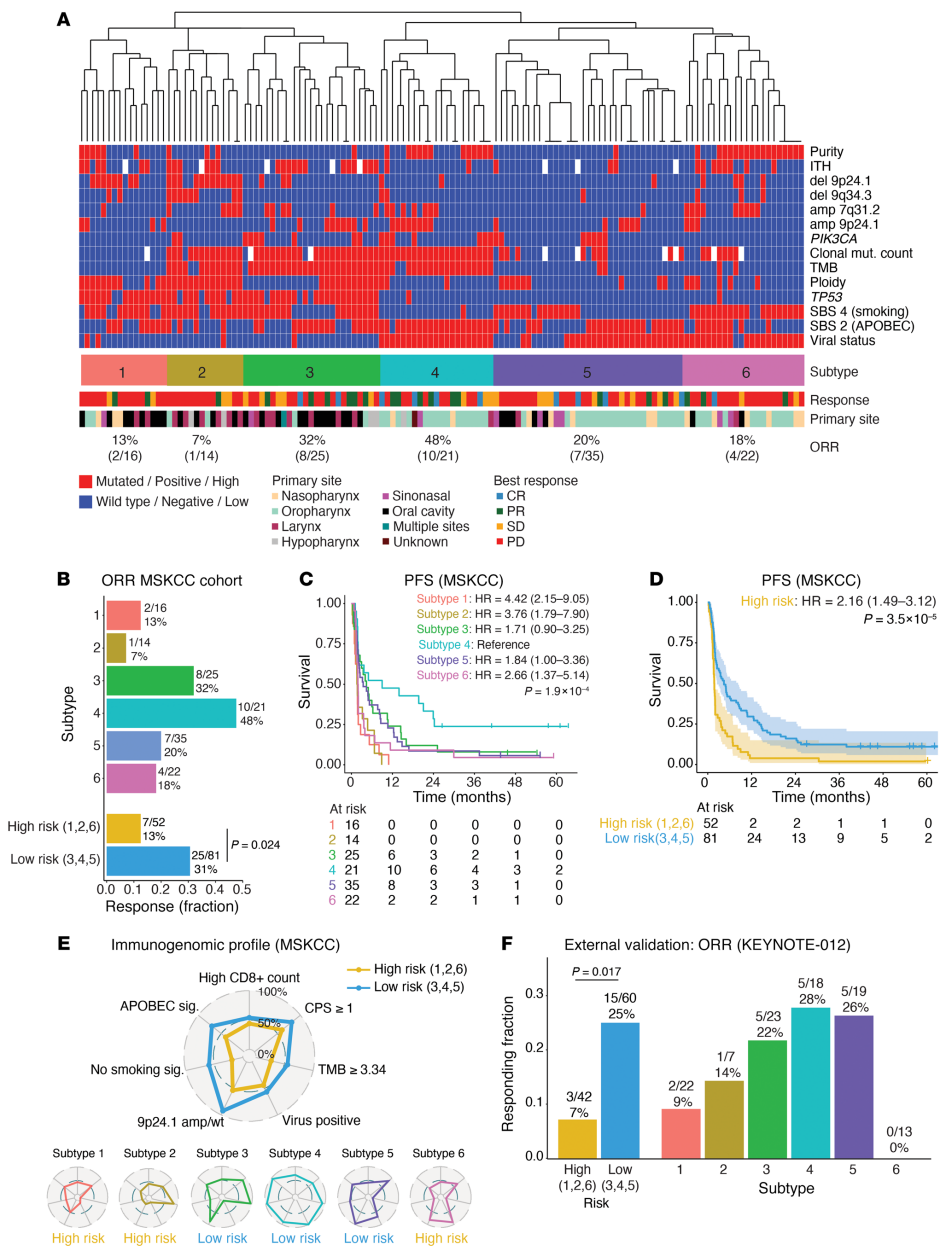
Molecular subtyping of HNSCC using WES data and its relevance to clinical outcomes after ICB treatment. (**A**) Hierarchical clustering of 133 R/M HNSCC samples based on 13 genomic features that significantly associated with PFS in a univariable Cox model (listed right) and viral status. The dendrogram was cut at constant height, yielding 6 subtypes. Bottom tracks show the ICB response (printed as a percentage) and the tumor site. (**B**) ORR per molecular subtype and for grouped subtypes considered high risk (subtypes 1, 2, and 6) and low risk (subtypes 3, 4, and 5). Total *n* = 133. The *P* value was calculated using Fisher’s exact test. (**C**) PFS estimate for each molecular subtype. HRs and 95% CIs were calculated using Cox regression, with subtype 4 as a reference. The *P* value calculated using a log-rank test. (**D**) PFS estimate for tumors belonging to subtypes considered high risk (1, 2, and 6) and low risk (3, 4, and 5). HRs and 95% CIs were calculated using Cox regression, with low-risk tumors as a reference. The *P* value was calculated using a log-rank test. (**E**) Immunogenomic profiles of high-risk (yellow) and low-risk (blue) samples as well as each subtype individually, based on 7 parameters: high CD8-positive T cell infiltration, CPS of 1 or higher, high TMB, viral positivity, absence of 9p24.1 deletion (locus of *CD274* [PD-L1], *PDCD1LG2* [PD-L2], and *JAK2*), absence of a smoking signature, and the presence of an APOBEC signature. Radars extend from 0%–100%; the percentage of tumors positive per parameter is shown. For CD8-positive T cells, the cohort median was used as a cutoff. Thresholds for TMB (3.34 muts/Mbp), APOBEC signature, and smoking signature were chosen to obtain the best performance for predicting PFS in a univariable model. Genomic variables were available for 133 samples and IHC features for 62 samples. amp/wt, amplified or wild-type (diploid) copy number. (**F**) External validation of the subtypes relevance to the ICB response using KEYNOTE-012 data on patients with HNSCC (*n* = 102). Bars represent the ORR. The *P* value was calculated using Fisher’s exact test.

We next investigated whether features in exome data could be used to classify patients into molecular subtypes relevant to immunotherapy response. First, we examined the association between the most prevalent genomic and molecular features (among the mutations, copy number alterations, mutational signatures, intratumoral heterogeneity [ITH], tumor purity, and viral status) and PFS and OS using univariable Cox regression in the V-negative and V-positive tumors, separately and combined, to identify features with potential predictive value. Ultimately, 14 features (13 genetic features and viral status) were selected and dichotomized into “low” or “high” categories on the basis of the threshold that maximally separated PFS. After performing unsupervised hierarchical clustering of these 14 features, we identified 6 distinct molecular HNSCC subtypes (Figure 3A). Subtypes 1–3 were generally *TP53*-mutant, V-negative tumors (13%, 7%, and 4%, respectively, were virus associated), whereas subtypes 4–6 were primarily *TP53* WT, V-positive (100%, 69%, and 91%, respectively).

The ORR for ICB treatment ranged from 7% (subtype 2) to 48% (subtype 4) (Figure 3B). In line with the ORR, significant differences in PFS were observed (*P* = 1.9 × 10^–4^) between the molecular subtypes — the median PFS was 9.1 months (95% CI: 2.0–∞) for subtype 4, and 1.7 (95% CI: 1.0–5.3) and 1.7 (95% CI: 1.4–6.8) months in subtype groups 1 and 2, respectively. HRs for subtypes 1 and 2 were 4.42 (95% CI: 2.15–9.05) and 3.76 (95% CI: 1.79–7.90), respectively, compared with subtype 4 (Figure 3C). Similar patterns were observed for OS curves per molecular subtype (*P* = 1.3 × 10^–4^) (Supplemental Figure 2A). On the basis of the ORR and PFS, we divided the 6 subgroups into low-risk (subtypes 3, 4, and 5) and high-risk (1, 2, and 6) categories, with differences in ORR, PFS, and OS (Figures 3, B and D, and Supplemental Figure 2B).

We profiled each subtype and risk category according to 7 variables previously described to correlate with ICB response: TMB, viral status, APOBEC signature, smoking signature, deletion of 9p24.1 (the locus of the CD247 [PD-L1], *PDCD1LG2* [PD-L2], and *JAK2* genes) (28, 29), CD8-positive T cell infiltration on IHC analysis, and PD-L1 CPS (Figure 3E and Supplemental Figure 2C) (15, 16, 29–33). As expected, the PD-L1 CPS was significantly lower in tumors with a 9p24.1 deletion (median 1.0 [IQR: 0.2–7.3]) compared with those without (5.1 [IQR: 1.4–45.0]; *P* = 0.039) (Supplemental Figure 2D).

The distribution of these features was more favorable in the low-risk tumors compared with high-risk HNSCCs (Figure 3E). The most favorable immunogenomic landscape was observed in subtype 4 (which had an ORR of 48% and the best PFS and OS). These tumors were defined by virus positivity, a high TMB, a strong APOBEC signature, an absent smoking signature, high CD8-positive T cell infiltration, and an absence of 9p24.1 deletion. Tumors in subtypes 1 and 2, conversely, had the lowest ORRs (13% and 7%) and the poorest PFS: they lacked viral antigens, had a poorly infiltrated T cell tumor microenvironment (TME), and a strong smoking signature. Subtype 1 also had a low TMB, whereas subtype 2 had higher a TMB but frequent (86%) 9p24.1 deletions. Subtypes 3, 5, and 6 had more intermediate immunogenomic characteristics. Subtype 3 was the only V-negative, smoking-associated subtype with a moderately high response rate to ICB (32%), potentially related to this subtype’s intact 9p24.1 with a higher CPS and a relatively high TMB with evidence of APOBEC-associated mutagenesis (34, 35). Subtype 5 (ORR, 20%) shared some of these features but included a minority (31%) of *TP53* WT, V-negative tumors, with a low TMB and more frequent smoking signature — perhaps explaining the more moderate response rate. Finally, subtype 6 (ORR, 18%) was composed of tumors with intermediate features — some favorable (V-positive, 9p24.1 mostly intact), and some unfavorable (heavy smoking signature, weak APOBEC signature, and low TMB), consistent with the observations of the adverse prognostic effect of smoking in HPV-associated tumors (2).

To validate these molecular subtyping results in an independent external data set, tumor and matched normal exome-sequencing data from 102 patients with R/M HNSCC treated with pembrolizumab in the KEYNOTE-012 trial were analyzed (15, 36). In the prespecified analysis plan, each sample was assigned to a molecular subtype (see Methods, Supplemental Figure 2E, and Supplemental Table 1) by an investigator blinded to the clinical outcome data. In line with the main cohort data, low-risk tumors (subtypes 3, 4, and 5; *n* = 60) in the KEYNOTE-012 validation data set had a significantly higher response rate than did high-risk tumors (subtypes 1, 2, and 6; *n* = 42): 25% vs. 7% (OR, 4.33 [90% CI: 1.44–13.02]; *P* = 0.017), with the highest ORR observed in subtype 4 (28%) (Figure 3F).

### Previously described biomarkers have modest predictive value for ICB treatment response in HNSCC.

**Figure 4:**
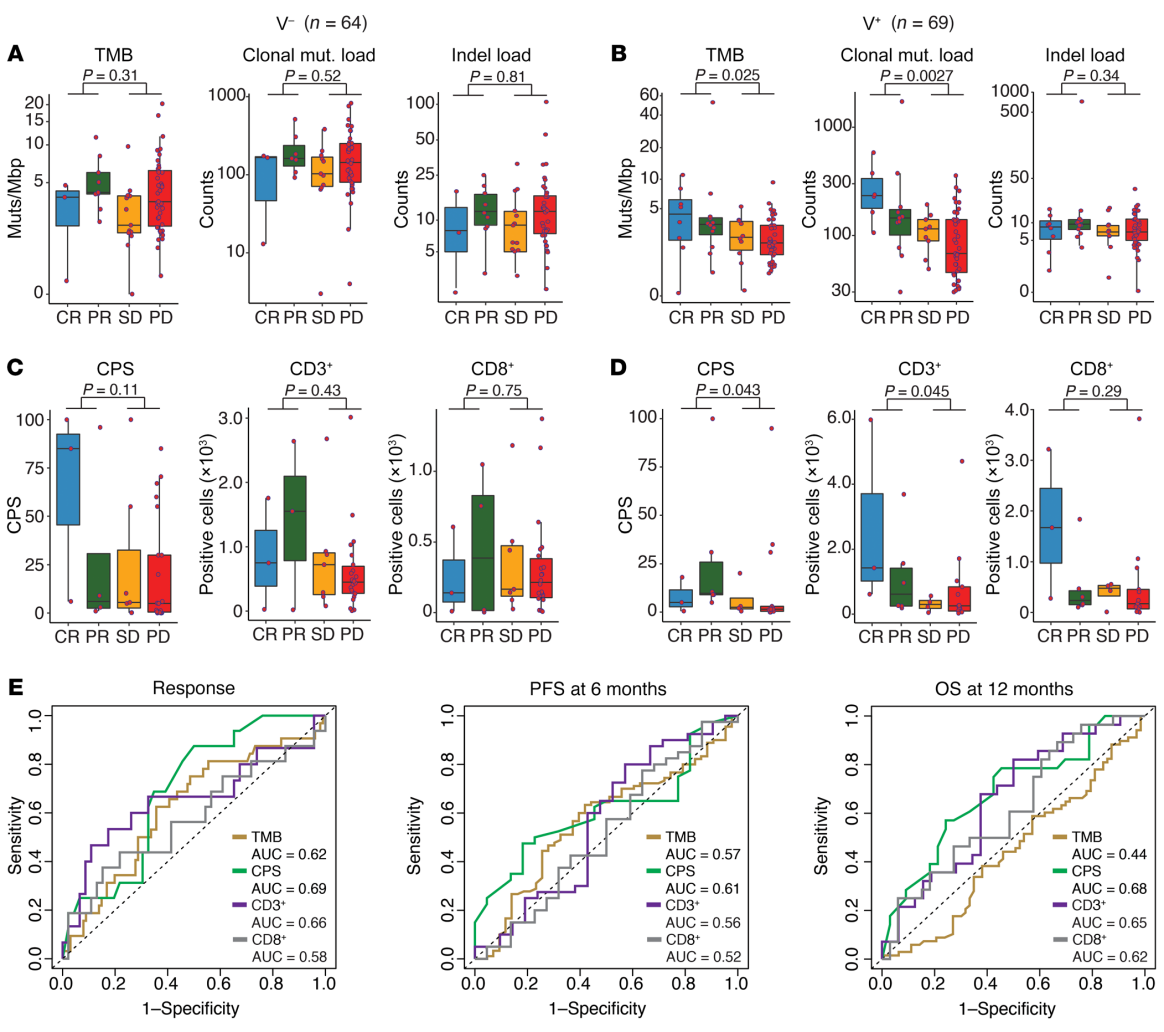
Association of previously described predictors of ICB treatment response in HNSCC. *P* values were calculated by comparing patients with a complete or partial response with patients who had stable or progressive disease using a Wilcoxon rank-sum test. Note that some *y* axes have been log_10_- or log_1p_-transformed for visualization purposes. (**A**) TMB, clonal mutational (mut.) load per exome, and indel load per exome in 64 V-negative samples, per objective response category. The clonal mutational load was available for 59 samples. (**B**) TMB, clonal mutational load per exome, and indel load per exome in 69 V-positive samples, per objective response category. The clonal mutational load was available for 65 samples. (**C**) CPS, intratumoral CD3-positive T cell count, and intratumoral CD8-positive T cell count in 36 V-negative samples, per objective response category. The CD3-positive T cell count was available in 35 samples. (**D**) CPS, intratumoral CD3-positive T cell count, and intratumoral CD8-positive T cell count in 26 V-positive samples, per objective response category. (**E**) ROC analysis illustrating the performance of the TMB, CPS, CD3-positive infiltration, and CD8-positive infiltration in predicting objective responses, 6-month PFS, and 12-month OS in the patients (V-negative and V-positive) for whom these data were available (*n* = 62). The AUROC curve (AUC) is printed in each plot.

Biomarkers previously described as having potential predictive value in HNSCC tumors treated with ICB were then explored: TMB (FDA-approved for the use of ICB in solid tumors), PD-L1 CPS, and intratumoral T cell infiltration (9, 14, 16). The median TMB was significantly higher in responding versus nonresponding patients with V-positive HNSCC (3.47 vs. 2.05 muts/Mbp; *P* = 0.025), but not in patients with V-negative HNSCC (4.08 vs. 3.32 muts/Mbp; *P* = 0.31) (Figure 4, A and B). More specifically, the association between TMB and ICB response seemed mainly driven by clonal mutations in the patients with V-positive HNSCC (median, 164 per exome in responders vs. 88 in nonresponders; *P* = 0.0027).

In the V-negative population, we found no statistically significant difference between responders and nonresponders with regard to the PD-L1 CPS (median, 9.0 vs. 5.0; *P* = 0.11), CD3-positive T cells (median, 1,150 vs. 466 cells/region of interest [ROI]; *P* = 0.43), or CD8-positive T cells (median, 140 vs. 202 cells/ROI; *P* = 0.75) (Figure 4C). However, in the V-positive subcohort, ICB-responsive patients had a significantly higher median CPS (9) and CD3-positive T cell infiltration (967 cells/ROI) compared with nonresponders (1.5 and 256 cells/ROI; *P* = 0.043 and 0.045, respectively), whereas the CD8-positive T cell abundance was not significantly different (median, 305 cells/ROI in responders vs. 242 in nonresponders; *P* = 0.29) (Figure 4D). Among the population of responders with V-negative or V-positive tumors, a greater fraction of the patients had a relatively high CPS (using various thresholds), but no statistically significant associations (Supplemental Figure 2F). Despite not performing well as a dichotomous variable, the overall performance of the CPS as a continuous variable was reasonable: area under the receiver operating characteristic (AUROC) values were 0.69 for response, 0.61 for 6-month PFS, and 0.68 for 12-month OS (Figure 4E).

### An integrated clinical-genomic model predicts survival after ICB treatment in HNSCC.

**Figure 5:**
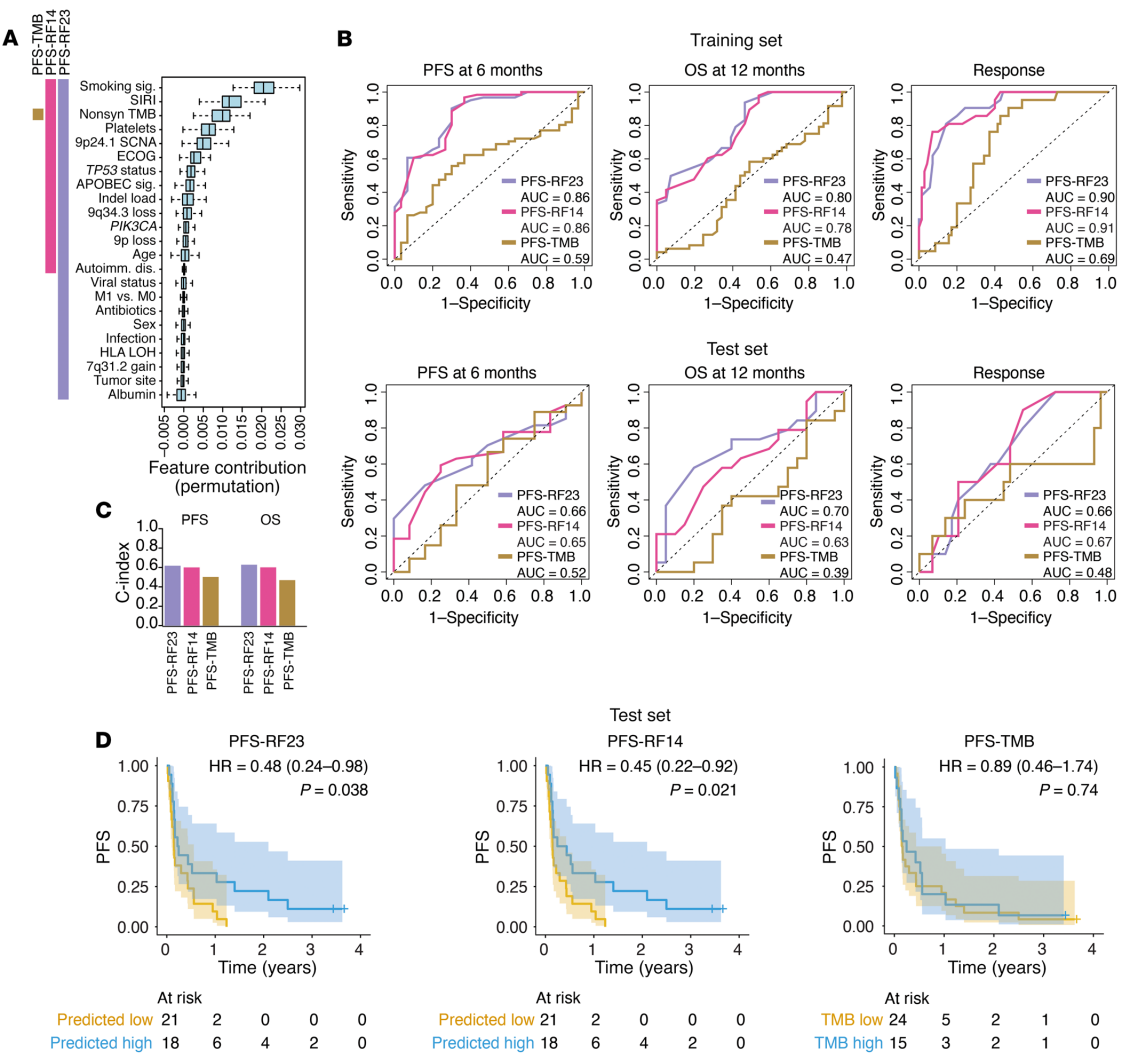
Training and testing of an integrated, clinical-genomic model in HNSCC using PFS as the outcome. (**A**) Feature contribution of 23 clinical and genomic variables to a RF classifier predicting PFS. Variables are ordered from highest to lowest feature contribution. Colored bars on the left indicate the variables included in the PFS-RF23 model (all), the PFS-RF14 model (top 14 variables only), and the PFS-TMB model (TMB only). Autoimmun. dis., autoimmune disease; nonsyn, nonsynonomous mutation. (**B**) ROC analysis illustrating the performance of the 3 models (PFS-RF23, PFS-RF14, and PFS-TMB) in predicting 6-month PFS, 12-month OS, and objective response in the 70% training set (top row of plots, *n* = 91) and 30% hold-out test set (bottom row, *n* = 39). Three patients were excluded due to incomplete clinical data. (**C**) Bar charts showing the C-index (38) for the PFS-RF23, PFS-RF14, and PFS-TMB model’s performance in predicting PFS and OS, calculated in the test set (*n* = 39). (**D**) Kaplan-Meier PFS analysis in the test set for the PFS-RF23, PFS-RF14, and PFS-TMB models. The median predicted PFS for each model was used as a threshold to divide patients into predicted high-survival (blue) and low-survival (yellow) groups. HRs and 95% CIs were calculated using Cox regression, with predicted low-survival tumors as a reference. *P* values were calculated using a log-rank test.

The predictive capability of the previously described biomarkers was modest, consistent with the hypothesis that the immunotherapy response is probably multifactorial and may not be sufficiently captured with 1 marker (37). Seeking to develop a clinical-genomic tool to predict tumor response with greater accuracy than existing biomarkers do, we tested and trained an ensemble random survival forest classifier to predict PFS. In addition to the genomic alterations derived from WES data, we added clinical variables, including patient age and performance status, tumor site and stage, infection and antibiotic use while on ICB, and values obtained from routine clinical laboratory blood testing. We first split the cohort into a training set (70%, *n* = 91) and hold-out test set (30%, *n* = 39; 3 patients were excluded because of incomplete clinical data), ensuring that the split was stratified to balance the distribution of key clinical and genomic variables in each of the sets. Twenty-three features (PFS-RF23) were selected and then calibrated in the training set with bootstrap aggregation (Figure 5A). The 14 features with an average permutation importance above 0 were selected to train a second, more parsimonious model (PFS-RF14) in the same training set. We then compared both RF models against a genomic biomarker of TMB alone in the hold-out test set, with 6-month PFS, 12-month OS, and objective response as endpoints. In the test set, the PFS-RF23 was able to better predict PFS, OS, and ORR (AUROC values of 0.66, 0.70, and 0.66, respectively), as was the PFS-RF14 model (0.65, 0.63, and 0.67), compared with the PFS-TMB model (0.52, 0.39, and 0.48) (Figure 5B). C-index values (38) in the test set were higher for PFS-RF23 and PFS-RF14 models compared with the PFS-TMB model (Figure 5C). The median predicted PFS for each model was then used as a threshold to divide patients into predicted high-survival and low-survival groups (Supplemental Figure 3A). In the test set, the PFS-RF23 (HR, 0.48 [0.24–0.98]; *P* = 0.038) and the PFS-RF14 model (HR, 0.45 [0.22–0.92]; *P* = 0.021) were significantly associated with PFS, while no significant association was seen with high TMB (HR, 0.89 [0.46–1.74]; *P* = 0.74) (Figure 5D). We obtained similar outcomes when analyzing OS as the clinical endpoint for high-survival and low-survival patients in these models trained on PFS (Supplemental Figure 3, B and C).

Following the same methodology and using the same 23 clinical and genetic variables, we trained 2 predictive models using OS as endpoint (OS-RF23 and OS-RF11), which were again compared with a TMB-based predictor (OS-TMB) (Supplemental Figure 4A). Analyses identical to the PFS model and setting PFS, OS, and ORR as endpoints yielded a similar predictive power (Supplemental Figure 4, A–D, and Supplemental Figure 5, A and B).

**Figure 6:**
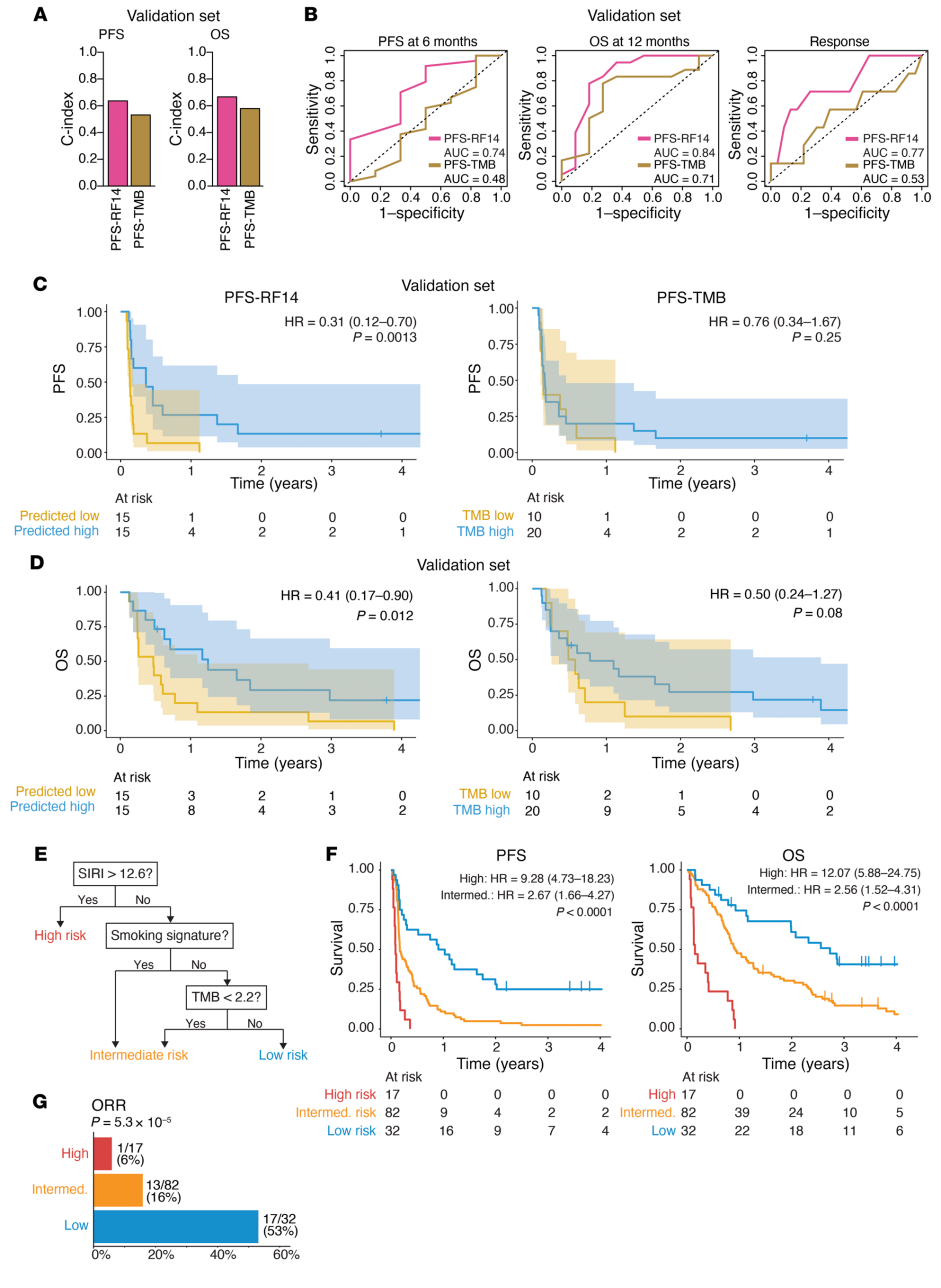
Validation of the clinical-genomic model associated with PFS upon ICB treatment in an independent cohort of 30 patients and model simplification using RPA. Patient characteristics for the independent cohort are provided in Supplemental Table 2, and model simplification using RPA is shown in Supplemental Figure 5. (**A**) C-index illustrating the performance of the PFS-RF14 model applied in the independent validation cohort (*n* = 30), compared with the model based on TMB (PFS-TMB). (**B**) ROC analysis illustrating the performance of the PFS-RF14 and TMB model in predicting 6-month PFS, 12-month OS, and objective response in the validation cohort (*n* = 30). (**C**) PFS in the validation cohort for the PFS-RF14 and PFS-TMB models. The median predicted PFS for each model was used to divide patients into predicted high-survival (blue) and low-survival (yellow) groups. HRs and 95% CIs were calculated using Cox regression, with predicted low-survival tumors as a reference. *P* values were calculated using a log-rank test. (**D**) OS in the validation cohort for the PFS-RF14 and PFS-TMB models. The median predicted PFS for each model was used as a threshold to divide patients into predicted high-survival and low-survival groups. HRs and 95% CIs were calculated using Cox regression, with predicted low-survival tumors (yellow) as a reference. (**E**) RPA classifier created in the main cohort (*n* = 131) using PFS as a dependent variable. Variables selected for the model were the top 3 features from the PFS-RF23 model: SIRI, TMB, and smoking signature. Patients were classified into high-risk, intermediate-risk, and low-risk groups. (**F**) PFS (left) and OS (right) of the high- (red), intermediate- (orange), and low-risk (blue) groups obtained using the RPA classifier in the main cohort (*n* = 131). HRs and 95% CIs were calculated using Cox regression, with low-risk tumors as a reference. *P* values were calculated using a log-rank test. (**G**) ORR in the high- (red), intermediate- (orange), and low-risk (blue) groups obtained using the RPA classifier in the main cohort (*n* = 131). The *P* value was calculated using a Fisher’s exact test with Freeman-Halton extension. Intermed., intermediate.

Finally, to validate the performance of the PFS-RF14 classifier in an independent cohort, we identified 30 additional patients with HNSCC (16 V-negative, 14 V-positive; see clinical data in Supplemental Table 2) treated at our center with anti–PD-1 drugs, who had complete clinical and genomic data available for analysis. These tumors were profiled with targeted next-generation sequencing (tNGS) using 3 versions of the MSK–Integrated Mutation Profiling of Actionable Cancer Targets (MSK-IMPACT) panel (39, 40) including 341 (3 samples), 410 (11 samples), or 468 genes (16 samples; genes per panel are listed in the Supplemental Supporting Data Values file). The PFS-RF14 model was chosen for validation because not all genomic features in RF23 were available from the tNGS panel; similarly, clinical smoking history was used instead of the smoking mutational signature. In this validation data set, we found that the PFS-RF14 model could predict PFS and OS (C-index, 0.63 and 0.67, respectively) better than the PFS model based on TMB (C-index, 0.53 and 0.58, respectively) (Figure 6A). The PFS-RF14 model outperformed TMB in predicting 6-month PFS (AUROC, 0.74 vs. 0.48), 12-month OS (0.84 vs. 0.71), and objective response (0.77 vs. 0.53) (Figure 6B). Finally, the patients in the independent cohort predicted to have a high PFS by the PFS-14 model (using the median as a threshold) had significantly longer PFS (HR, 0.31 [0.12–0.70]; *P* = 0.0013) and OS (HR, 0.41 [0.17–0.90]; *P* = 0.012) than did those in the low PFS group, while the prediction based on TMB yielded no statistically significant separation of PFS (HR, 0.76 [0.34–1.67]; *P* = 0.25) or OS (HR, 0.50 [0.24–1.27]; *P* = 0.08) (Figure 6, C and D). The low predictive value of TMB in the validation cohort is consistent with prior literature; for example, a nonsignificant association with OS after ICB therapy (HR, 0.76 [0.33–1.76]) was found in a prior study of 174 patients with HNSCC (9).

To evaluate whether these predictive models also convey prognostic information outside of the context of ICB treatment, we tested the RF14 model in a cohort of patients with HNSCC never treated with immunotherapy. In predicting this cohort’s OS, the C-index and AUROC for the RF14 model were higher than for TMB alone (Supplemental Figure 5, C and D). In this cohort, a high TMB (HR, 2.74; *P* = 0.028) and the RF14 model (HR, 0.4; *P* = 0.037) were significantly associated with OS (Supplemental Figure 5, E and F), indicating that the RF14 model also had prognostic power, albeit slightly less than the power of RF14 to predict ICB treatment outcomes.

Having validated the performance of the PFS-RF14 model in the hold-out test set and the independent validation set, we sought to develop a more parsimonious model that might be more feasible for clinical use. A recursive partitioning analysis with PFS as a dependent variable selected the top 3 features from the RF model: systemic inflammatory response index (SIRI) (the ratio of [neutrophils × monocytes]/lymphocytes), TMB, and smoking mutational signature. These 3 features categorized patients with HNSCC into low-, intermediate-, and high-risk groups (Figure 6E). Intermediate- and high-risk patients had a significantly inferior PFS (HR, 2.67 [1.66–4.27] and 9.28 [4.73–18.23], respectively) and OS (HR, 2.56 [1.52–4.31] and 12.07 [5.88–24.75]) compared with low-risk patients (both *P* < 0.0001) (Figure 6F). The ORR was also significantly higher in the low-risk (53%) compared with the intermediate- (16%) and high-risk (6%; *P* = 5.3 × 10^–5^) groups (Figure 6G). This 3-feature recursive partitioning analysis (RPA) classifier was additionally tested in the tNGS IMPACT validation cohort (*n* = 30), again using clinical smoking history as a proxy for the smoking mutational signature, where it accurately predicted patients at high risk for poor OS, although the association with PFS was not statistically significant in this small cohort (Supplemental Figure 5, G and H).

These models demonstrate that the combination of clinical and genomic data can generate a model that predicts PFS, OS, and response in patients with HNSCC treated with ICB, and with greater accuracy than using TMB alone. A simpler, 3-feature model incorporating only SIRI, TMB, and smoking signature was able to classify patients into low-, intermediate-, and high-risk groups with good discrimination of PFS and OS, although further validation is required.

## Discussion

Here, we report our analyses of 133 patients with R/M HNSCC treated with anti–PD-1/anti–PD-L1 ICB, including data on high-depth WES, clinical features, and routine laboratory tests. To our knowledge, this is the largest and most comprehensive data set reported to date. We defined molecular subtypes of HNSCC relevant to ICB response and constructed and validated a model that effectively predicted survival and response with greater accuracy than the existing FDA-approved biomarker of TMB.

We report a 24% ORR and found that patients with virus-associated tumors (V-positive) had a numerically superior ORR (29% vs. 19%; *P* = 0.22) and PFS (HR, 0.72 [95% CI: 0.50–1.03]; *P* = 0.065) and significantly longer OS (HR, 0.55 [95% CI: 0.37–0.80]; *P* = 0.0015) compared with V-negative patients. These results agree with previous observations from a pooled analysis of 3 clinical trials (41), but the association between virus positivity and ICB response in HNSCC remains incompletely clarified.

This study confirms the distinct genomic profiles of V-negative and V-positive HNSCCs (21) and demonstrates a high prevalence of a smoking signature (76%), *TP53* mutations (67%), and a hyperploid copy number (59%) in V-negative tumors. We identified a strong enrichment of *TERT* promoter mutations in oral cavity tumors. We found that TMB alone was a fairly weak biomarker of ICB response in HNSCC — illustrating that considerable mutational antigenicity alone is not always sufficient to develop a response. It may be that not only the total TMB, but also the origin of mutations, plays a role in ICB response in HNSCC. This is supported by the association of a smoking mutational signature — frequently seen in V-negative tumors — with nonresponses in this cohort, which could be related to our previously observed association between a smoking signature and a more immunosuppressive microenvironment in HNSCC (31). Furthermore, the high prevalence of a mutational signature associated with APOBEC enzymatic activity in V-positive tumors (70%) — positively associated with immune infiltration and neoantigen load in HNSCC (32) — may have contributed to the higher ORR in V-positive tumors.

The multitude of factors involved in antitumor immunity (37) makes it unlikely that 1 single biomarker will suffice to predict ICB outcomes, a notion that we underline by demonstrating the relatively modest individual performance of TMB, PD-L1 CPS, and CD8-positive T cell infiltration as biomarkers. Our clustering analysis highlights the fact that HNSCC may be classified into 6 different molecular subtypes with distinct outcomes, based chiefly on *TP53* mutation status (highly correlated to tumor viral status), TMB, 9p24.1 deletion, and sample tumor purity. Notably, the prognostic association of the molecular risk classification was validated in an independent, external cohort of 102 patients with R/M HNSCC. Furthermore, the distinct immunogenomic profiles of higher- and lower-risk tumors — the latter of which are more often characterized by high CD8-positive T cell infiltration, a CPS of 1 or higher, a high TMB, an APOBEC signature, viral positivity, a weaker smoking signature, and a genetically intact PD-L1 pathway — seemingly confirm the collective importance of these factors to ICB response in HNSCC.

To develop a predictive model, we trained a random survival forest model integrating clinical and genetic data. In the hold-out test data set, we found this model able to predict treatment response and survival in patients with HNSCC with greater accuracy than TMB alone. The robustness of this model was indicated by a similar performance in an independent data set for which only tNGS data were available. We attempted to make this model more intuitive and broadly accessible by showing that patients might be accurately stratified into high and low PFS groups by using just 2 WES-derived features (smoking signature and TMB) — both of which could potentially be derived from relatively low-cost tNGS panels — and 1 clinical laboratory variable (SIRI). Although this model showed accurate performance in our validation data set, we caution that a more precise quantification of the accuracy of this model will require application in a broader number of R/M HNSCC cohorts drawn from diverse clinical settings.

Biomarkers can convey both prognostic and predictive information at the same time (e.g., HPV status) — they are not mutually exclusive terms (42). Our models have predictive value, as indicated by the association with the probability of a tumor response. Interestingly, these models also have some prognostic value, as they are associated with OS in non-ICB-treated HNSCC patients. In the non-ICB-treated cohort, high TMB had a strong negative prognostic effect, directionally opposite to its positive predictive value in patients who were treated with ICB, which is in line with our previous findings in a pan-cancer cohort (43).

This study is inherently limited by its retrospective design. The molecular subtyping analysis was validated in an external, independent data set. In addition, the integrated clinical-genomic predictive model and the simplified 3-feature classifier (SIRI, TMB, smoking signature) were validated in a hold-out test set and an additional independent validation data set. Still, we believe that an even better test of clinical utility and generalizability will be the application of these markers in a prospective clinical cohort, which is the intended next step of this research. To this end, we propose to test the RF23 or RF14 model when all relevant data are present, and to use the 3-feature RPA classifier if the available data are more limited. In addition, the limited availability of tumor material precluded immunohistochemical characterization of the TME in approximately half of this cohort. It may be that further integration of tumor genomic data with microenvironmental and transcriptomic data will yield more accurate predictive models. Finally, we cannot rule out a potential effect of radiotherapy administered while the patient is on ICB treatment (*n* = 40, 30%). However, we opted to include these patients because (a) we could adjudicate the ORR independently (see Methods), and (b) we aimed for our cohort to reflect real-world practice, in which palliative radiotherapy is often used for local symptomatic control, and excluding these patients would bias our cohort by enriching for more indolent tumors.

In conclusion, we report on a large cohort of patients with R/M HNSCC treated with ICB. WES data revealed 6 molecular HNSCC subtypes with distinct prognostic profiles, a finding reproduced in a large external cohort. Finally, we used combined clinical and genomic data to train a model that — upon further validation in external data sets — may be used to guide researchers and clinicians looking for a more effective and personalized way to use ICB agents for the treatment of HNSCC.

## Methods

### Cohort characteristics.

The main cohort patients had R/M HNSCC and received at least 1 dose of anti–PD-(L)1 and/or anti–CTLA-4 ICB at MSKCC after 2015, when the collection of tumor tissues and blood for DNA extraction and genomic profiling was part of routine clinical care. All patients provided written informed consent for tumor genomic sequencing. Patients treated with ICB as neoadjuvant or adjuvant treatment and patients with cutaneous squamous cell carcinoma (SCC), salivary gland cancers, or thyroid tumors were excluded. In total, 133 patients with R/M HNSCC patients — treated at MSKCC and with biospecimens available for WES — were identified.

An additional cohort with the same inclusion criteria, but for whom WES was not performed, had genomic data available from tNGS as part of clinical care using the MSK-IMPACT platform (*n* = 30, Supplemental Table 2). These patients also provided written informed consent for tumor sequencing. This data set was used for validation.

The tumor sample obtained on the date closed to the start of ICB was selected for tNGS or WES in both cohorts. For 18 WES (13.5%) and 11 MSK-IMPACT patients (36.7%), only samples obtained after the start of ICB were available.

Data from a cohort of patients with HNSCC treated with immunotherapy, who had WES performed, and for whom response data were available from the KEYNOTE-012 study served as an external validation data set (15, 36). All 107 samples from KEYNOTE-012 HNSCC B1 and B2 cohorts were analyzed, but only samples that passed quality control metrics and had complete data for our study were used (*n* = 102).

We assembled a cohort of 65 patients with HNSCC who were diagnosed and/or treated at our center and had MSK-IMPACT data available, but never received immunotherapy. The percentage of V-positive tumors was similar to the percentage in the main cohort (42% vs. 52%).

### Outcomes.

Clinical records were reviewed for the primary study outcomes: ICB response, PFS, and OS. The first line of ICB was used to annotate patients who received multiple lines of ICB. Objective response was assessed using RECIST, version 1.1(17). If formal RECIST reads were unavailable (*n* = 72, 54%), physicians’ notes and imaging studies were reviewed using the same criteria. For consistency, all patients were reviewed by the same investigator (and audited by a senior author). An objective response was defined as a complete or partial response. Clinical benefit was defined as an objective response or stable disease lasting at least 6 months. PFS was defined as the time from the first ICB infusion to disease progression or death of any cause; patients without progression were censored at their last appointment. OS was defined as the time from the first ICB infusion to death of any cause; patients alive at the time of the review were censored at their last contact. For the cohort of patients never treated with immunotherapy, the outcome of interest was OS calculated from the start date of the first treatment or, if only supportive treatment was administered, the date of diagnosis.

Patients treated with radiotherapy while on ICB (*n* = 40, 30%) were only classified as responders if there was a response in nonirradiated lesion(s) (*n* = 8) or if the response occurred before radiotherapy was commenced (*n* = 4). Of the 19 patients (14%) who received another systemic treatment while on ICB (detailed in Supplemental Table 3), 10 were treated with cytotoxic chemotherapy. Four patients (all nonresponders) received chemotherapy concurrently with the start of ICB. Six patients received chemotherapy initiated after the start of ICB treatment: 5 patients without a response and 1 patient with a partial response that occurred during ICB therapy alone, before the start of chemotherapy.

### HPV and EBV detection.

HPV status was determined through chart review and integration of available assays: p16 IHC, PCR, and DNA or RNA ISH. If only 1 test was performed, and it was positive, the tumor was categorized as HPV-positive. If more than 1 test was performed, a tumor was categorized HP-positive only if all the tests performed were positive, with 1 exception: a tumor was categorized as HPV-positive if DNA ISH was negative, provided either p16 IHC or RNA ISH was positive. EBV status was determined using Epstein-Barr virus–encoded RNAs (EBER) ISH.

### IHC.

Tissue was available for analyses of the TME using IHC in 62 cases (47%). In the remaining cases, additional tissue was unavailable, generally because of sample exhaustion. The tissue sample selected for IHC was the closest prior to the start of ICB, and in 1 patient (<1%) a post-ICB sample was used. The primary antibodies used were as follows: CD3 (clone LN10, dilution 1:100, Leica Biosystems), CD8 (clone 4B11, ready-to-use, Leica Biosystems), and PD-L1 (clone E1L3N, dilution 1:400, Cell Signaling Technology). The E1L3N clone, validated at our center, generates CPS results highly comparable to those of the 22C3 clone in HNSCC (44, 45). The PD-L1 CPS was evaluated on whole slides by a head and neck pathologist and defined as follows: (number of PD-L1–positive tumor and immune cells)/(total number of tumor cells) × 100. The whole slides stained for CD3 and CD8 were scanned using Mirax (3DHISTECH). After scanning, each whole slide was reviewed, and 3 circular intratumoral areas (ROIs) measuring 1 mm in diameter were selected using ImageJ (NIH) (also used for quantification). Thresholding was performed on brown (positive cells marked with DAB) and blue (hematoxylin-labeled nuclei) areas. Automatic enumeration of the total number of CD3-positive or CD8-positive cells in each ROI was obtained using specific scripts for each stain (see Supplemental Figure 2, C and D). The arithmetic mean of the cell counts in the 3 ROIs per sample was used for further analysis.

### WES.

Pre-enrichment libraries were created using the Illumina TruSight Oncology DNA Library Prep Kit or the KAPA HyperPrep Kit (Roche) with 40 ng or 200 ng input DNA per sample, respectively. KAPA libraries were purified and quantified with a Qubit dsDNA High Sensitivity assay (Thermo Fisher Scientific), and 500 ng was used for enrichment. TruSight Oncology index PCR products were directly used for enrichment following the TruSight Oncology Reference Guide. Target enrichment was performed using the Illumina TruSight Oncology Enrichment Kit with Integrated DNA Technologies (IDT) xGen Universal Blockers and the IDT xGen Exome Research Panel. A single hybridization was done overnight at 62°C. Post-enrichment libraries were normalized using bead-based normalization and then pooled. Samples were sequenced with 151 bp paired-end reads on the Illumina NovaSeq 6000 S4 flow cell using the XP workflow for individual lane loading with 12 libraries per lane. On average, each sample yielded 612 million reads and a median target coverage depth of 776×. Samples with a depth of coverage of less than 150× were excluded from this study.

### Variant calling and TMB.

WES reads were aligned using the Burrows-Wheeler Aligner (BWA-MEM) with the Sequence Alignment/Map (SAMtools) utility to align DNA sequences in FASTQ files to the hg19 genome. We used Strelka-2.9 (46) to perform small variant calling on paired tumor-normal BAM files for each sample after removing duplicate reads. Low-confidence single nucleotide variants (SNVs) were removed using the following criteria: tumor VAF≥0.05, DP.tumor≥50, DP.normal≥20, AD.tumor≥5, and VAFnormal/VAFtumor<0.2. Only variants called on both strands were called as high-confidence ones. The TMB was calculated as the total number of somatic mutations normalized to the exonic coverage in megabases. SciClone (version 1.1) (47) was implemented to calculate the cancer cell fraction (CCF). The clonal mutational load was calculated by counting mutations with a CCF of greater than 0.5 (48). Sequenza (version 2.1) (49) was used to estimate tumor purity and ploidy.

### Copy number alteration and HLA LOH.

Copy numbers were estimated using CNV Robust Analysis For Tumors (CRAFT) (50). CRAFT determined the read coverage of each amplicon or “bin” for the sample using a set of baseline samples as input. Then, a sample’s bin count was modeled as a linear combination of baselines, and the model prediction was used as a baseline-corrected value. Next, the effects of GC bias were removed using GC quantile normalization. Gene amplification or deletion events were determined using empirically determined cutoff values. We first defined the probability of deletion or amplification at the arm level as the total number of deleted or amplified genes divided by the total number of genes. Then, we considered an arm to be deleted or amplified if the probability of deletion or amplification exceeded 20%. Allele-specific HLA LOH data were obtained using FACETS (51).

### Mutational signature analysis.

Mutational signature analysis was done using maftools (52) following the authors’ recommendations. Briefly, trinucleotide frequencies were extracted, after which mutational signatures were obtained using non-negative matrix factorization. The top 4 signatures were chosen by inspecting the cophenetic metric and cosine similarity index with COSMIC SBS (23) signatures used for annotation.

### Molecular subtype classification.

We first evaluated whether each genomic feature with potential predictive value based on a literature review could predict PFS in the V-positive group, the V-negative group, or the whole cohort, by dichotomizing the continuous values into low and high. The threshold for each feature was chosen to attain the best performance in predicting PFS in a univariable survival model. Features with a *P* value of greater than 0.1 were not considered potentially predictive and were excluded. Next, we performed unsupervised hierarchical clustering of the 13 features that remained and of viral status. The hierarchical tree was cut at a constant height to obtain 6 distinct clusters. Subtypes were categorized into high-risk (subtypes 1, 2, and 6) and low-risk (subtypes 3, 4, and 5) groups on the basis of the differences in the ORR and PFS.

To facilitate the validation of the clinical utility of our molecular subtyping in an independent cohort (15, 36), we defined a rule-based classifier by characterizing the most (or least) enriched feature per cluster. We used recursive partitioning and regression trees to train a rule-based classifier using the same molecular features derived from WES minus viral status. Among the features, only *TP53* mutation status, TMB, 9p24.1 deletion, and tumor purity were informative. The following logic was used to classify all samples into molecular subtypes 1–6. (a) *TP53* status (mutated or WT) was used to assign the sample to subtype 1, 2, or 3 versus subtype 4, 5, or 6 (first hierarchy). (b) In the second hierarchy, we could best distinguish subtypes 1, 2, and 3 by 9p24.1 deletion (dominant feature of subtype 2) and TMB status (based on enrichment of TMB-low in subtype 1 vs. subtypes 2 and 3). (c) High tumor purity was the principal characteristic of subtype 6 compared with subtypes 4 and 5, whereas a high TMB could distinguish subtype 4 from subtypes 5 and 6.

Using only 4 features, the resulting classifier accurately reconstructed the subtypes assigned using hierarchical clustering with approximately 86% concordance in the high-risk (subtypes 1, 2, and 6) versus low-risk (subtypes 3, 4, and 5) classifications (Supplemental Figure 2F and Supplemental Table 1).

### WES analysis of the KEYNOTE-012 external validation cohort.

Raw paired tumor and normal WES data from the KEYNOTE-012 HNSCC study (15) were downloaded and processed at MSKCC using an independent pipeline that was previously described in detail (53). In brief, the sequences were aligned to the b37 reference genome (GATK bundle, version 2.3) using BWA-MEM (version 0.7.15-rl1140) (54). Aligned BAM files were further marked for duplicates and underwent indel realignment using GATK (version 3.7). Alignment details can be found at: https://github.com/jrflab/modules/blob/master/aligners/bwamemAligner.mk

Somatic SNVs were called using MuTect (version 1.1.7) (55). Indels were called with Strelka (version 1.0.15) (56), VarScan2 (version 2.3.7) (57), Platypus (version 0.8.1) (58), and Scalpel (version 0.5) (59). Indels called by 2 or more callers were included. Details on this pipeline can be found at: https://github.com/jrflab/modules/tree/master/variant_callers/somatic Somatic copy number alterations and LOH data were obtained using FACETS (version 0.5.6) (51). Arm-level copy numbers were calculated by taking the average segment copy number on the arm, weighed by the segment length. Gene-level copy number changes were determined by whether the total copy number of the gene was greater (gain) or lower (loss) than the average ploidy of the tumor as calculated by FACETS. Homdels were determined when the gene’s median log-ratio (LR) was less than the median segment LR value subtracted by 2.5 times the SD of the segment LR.

### Comparison of the KEYNOTE-012 external validation cohort and the main cohort.

Of the 107 samples in the Keynote-012 HNSCC cohort, 106 tumor-normal pairs passed WES quality control with confirmed tumor-normal matching. In 3 samples, purity could not be determined by FACETS, precluding molecular subtype classification. In 1 sample that was hypermutated because of a mutation in *POLE*, predicting a very high probability of the ICB response (60), the molecular subtype could not accurately be assigned in the absence of similarly hypermutated tumors in the main cohort. The final KEYNOTE-012 validation cohort thus consisted of 102 patients.

Quantile normalization mapped TMB of fewer than 3.34 muts/Mbp in the MSKCC cohort to TMB of fewer than 90 muts/exome in the KEYNOTE-012 cohort. With blinding to the clinical data, the KEYNOTE-012 patients were classified into molecular subtypes 1–6 using the 4-feature classifier described above. In consideration of the small number of patients in the subgroups and multiple hypothesis testing, the preplanned analysis in this validation data set allowed for statistical comparison of clinical outcomes only between low-risk and high-risk tumors.

### Random forest classifier.

Three patients from the main cohort (*n* = 133) had missing clinical data and were excluded. The 130 patients were split into a training set (70%) and a test set (30%). The imbalance score between the training and test sets was measured as the sum of the difference in medians (continuous variables) or the sum of the difference in frequency (categorical variables) for the most prevalent genomic alterations. This process was repeated 1,000 times, after which the split with the lowest imbalance score was selected, ensuring that the training and test sets were similarly sampled from the same distribution.

Then, in the training set (*n* = 91), we performed univariable analyses with PFS as the outcome using the V-positive group, the V-negative group, and both groups combined, after which we selected features that had a *P* value of less than 0.2 (Supplemental Table 4). Observed mutations, copy number alterations, and clinical features considered clinically relevant or with a previously shown association with outcomes were added.

After removing features with missing values, 23 features were considered for use to train a random forest (RF) classifier. We used a survival RF classifier model implemented in the R randomForestSRC package (61) with ntree=100 and block.size=1 to train a classifier on PFS or OS on training set patients (RF23). Training was performed 1,000 times, and the average statistics were reported. Permutation variable importance was measured and used to choose the most predictive features. Briefly, 14 features from the model trained on PFS (11 features in the model trained on OS) with average positive permutation importance were kept to retrain a more parsimonious model named RF14 (and RF11 for the OS model). Test set patients with a predicted survival above or below the median were defined as the high-survival or low-survival group, respectively. Model performance was assessed in the training and test sets using the C-index (38) and time-dependent ROC plots for 6-month PFS and 12-month OS. Furthermore, Kaplan-Meier survival analyses were performed in the test set, in which patients predicted to have low survival were compared with those with a predicted high survival.

We used the independent MSK-IMPACT tNGS cohort (*n* = 30) to validate the RF14 model. The MSK-IMPACT panel covers limited regions of the genome, precluding accurate identification of mutational signatures. Furthermore, the INDEL load was not reported. Therefore, values for the APOBEC signature and INDEL load were estimated using imputation as provided in the randomForestSRC package. A clinical smoking history was used as a proxy for the smoking mutational signature.

### RPA.

A simpler model was obtained after the RF using RPA with PFS as a dependent variable in the main data set (*n* = 131; *n* = 2 patients excluded because of incomplete data). The variables selected for the model were the top 3 features from the RF: neutrophils × monocytes/lymphocytes (known as the SIRI), TMB, and the smoking mutational signature. The model also included viral status to control for a possible bias. The RPA retained all the top 3 features from the RF model but did not retain viral status. Four final nodes were obtained, but 2 had a similar HRs and were merged. As such, 3 final groups (low-, intermediate-, and high-risk) were established. Validation was performed using the MSK-IMPACT tNGS cohort. As described above, a clinical smoking history was used for these patients instead of the smoking mutational signature.

### Statistics.

Box plots were used to show the distributions of continuous variables. The horizontal line represents the median and boxes the IQR. Whiskers extend from Q1 and Q3 to the minimal and maximal values, but no further than 1.5 × IQR. Survival curves (PFS and OS) were estimated using Kaplan-Meier methodology. *P* values of less than 0.05 indicate statistical significance. *P* values for Kaplan-Meier analyses were derived using the log-rank test. HRs and 95% CIs were calculated using a univariable or multivariable Cox proportional hazards model. The heatmap dendrogram was obtained using the heatmap.2 function from the gplots library in R at default settings. Radar plots were based on 7 parameters suggested as positively associated with the ICB response: high CD8-positive T cell infiltration, a CPS of 1 or higher, high TMB, virus positivity, absence of a 9p24.1 deletion, the absence of a smoking mutational signature, and the presence of an APOBEC signature. All points on the radars extend from 0% to 100%, and the fraction of tumors positive for each parameter is shown. For CD8-positive T cell infiltration, the cohort median was used as a cutoff to define a “high” count. The thresholds for TMB (3.34 muts/Mbp), APOBEC signature, and smoking signature were chosen to obtain the best performance for predicting PFS in a univariable survival model. Where appropriate, median values were compared across groups, and *P* values were calculated using a Wilcoxon rank-sum test (in the case of 2 groups) or a Kruskal-Wallis test (in the case of >2 groups). Proportions were compared across groups using a Fisher’s exact test, and the Freeman-Halton extension was applied when more than 2 groups were compared. All reported *P* values are 2 sided, except when a directional hypothesis was tested in the validation sets (i.e., KEYNOTE-012 or IMPACT), for which a 1-sided *P* value is reported. As our focus was not to identify new features of the well-described genomic landscape of HNSCC, we only performed limited hypothesis testing among a limited number of mutations (TERT promoter) (62, 63), copy number variants (aneuploidy) (64), 9p (65, 66), HLA LOH (27), and mutational signatures (smoking, APOBEC) (67–69), all of which were previously suggested to be of interest. Hence, we did not perform broad multiple hypothesis testing across the genome and report nominal *P* values for these comparisons. Stata Statistical Software, Release 16 (StataCorp), and R, version 3.5.1, were used.

### Study approval.

This retrospective study was approved by the IRB of MSKCC.

### Data availability.

All deidentified WES data have been deposited in the Sequence Read Archive (SRA) (https://www.ncbi.nlm.nih.gov/bioproject/PRJNA820894/; accession no. PRJNA820894). All deidentified clinical, genomic, and immunohistochemical data that underlie the results presented in this manuscript have been publicly deposited on Zenodo (https://doi.org/10.5281/zenodo.8051754). Values for all data points shown in graphs are provided in the Supplemental Supporting Data Values file.

## Author contributions

CV and LGTM conceptualized the study. CV, JLV, MG, BX, SK, and LGTM acquired, analyzed, and interpreted data. CV, JLV, MG, and LGTM performed statistical analyses. CV, JLV, MG, and LGTM wrote the original draft of the manuscript. All authors reviewed and edited the manuscript. JLV and MG conducted visualization analyses. LGTM, LL, and TP supervised the study. LGTM acquired funding for the project. CV, MG, and JLV contributed equally to the work and share first authorship, and the first-listed name was rotated across academic presentations and the published manuscript, and, accordingly, CV, MG, and JLV agree and assert that any permutation of the order of these names is correct and acceptable. AP, CF, CYH, DC, ESK, ML, NR, RS, and XP performed specialized clinical or genomic analyses. ALH, CLZ, DBS, DC, LAB, NR, and TAC contributed oncologic, genomic, and/or statistical review and expertise.

## Supplementary Material

Supplemental data

ICMJE disclosure forms

Supporting data values

## Figures and Tables

**Table 2:**
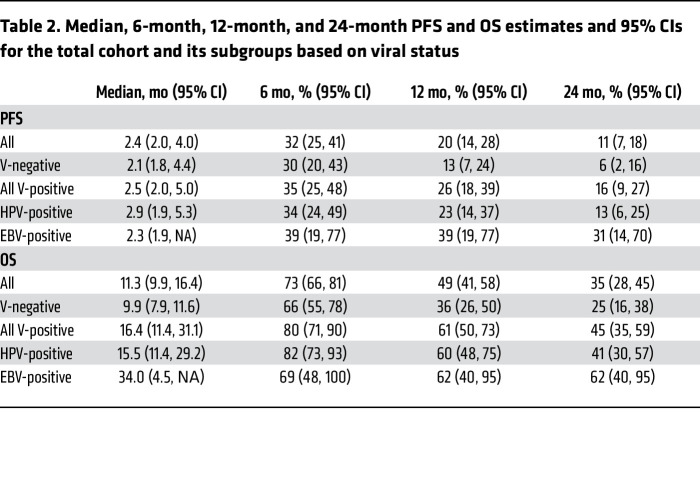
Median, 6-month, 12-month, and 24-month PFS and OS estimates and 95% CIs for the total cohort and its subgroups based on viral status

**Table 1:**
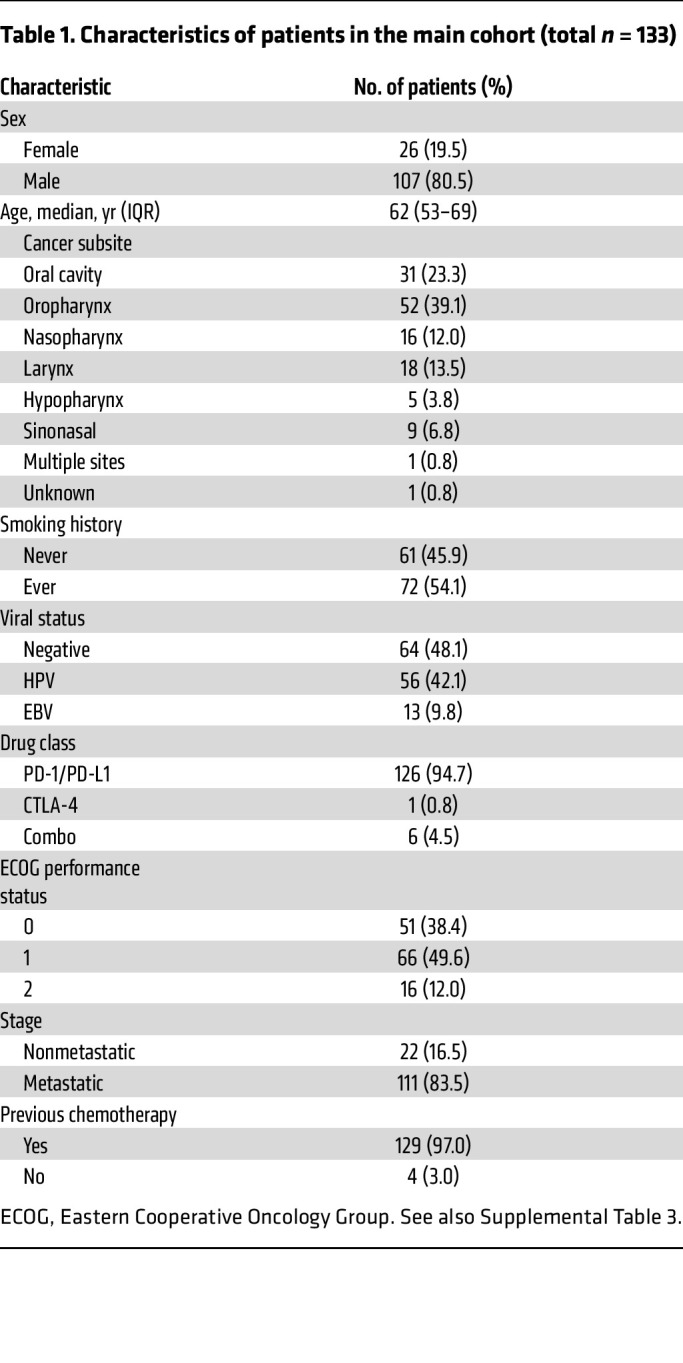
Characteristics of patients in the main cohort (total *n* = 133)

## Version 1 08/10/2023

In-Press Preview

## Version 2 10/02/2023

Electronic publication
