# Supplementary material for: Clinical-genomic determinants of immune checkpoint blockade response in head and neck squamous cell carcinoma
Source: J Clin Invest. 2023 Oct 2;133(19):e169823. doi: 10.1172/JCI169823 (PMC10541199; doi:10.1172/JCI169823)
Supplement: Supplemental data [file jci-133-169823-s080.pdf]

Clinical and genomic determinants of the efficacy of immune checkpoint blockade therapy in head and neck squamous cell carcinoma — Supplemental Material

Cristina Valero[1#], Mahdi Golkaram[2#], Joris L. Vos[1#], Bin Xu[3], Conall Fitzgerald[1], Mark Lee[1], Shannon Kaplan[2], Catherine Y. Han[1], Xin Pei[4], Reith Sarkar[4], Lillian A. Boe[5], Abhinav Pandey[1], Elizabeth S. Koh[1], Charlotte L. Zuur[6,7], David B. Solit[8], Traci Pawlowski[2], Li Liu[2], Alan L. Ho[8], Diego Chowell[9], Nadeem Riaz[4], Timothy A. Chan[10], Luc G.T. Morris[1*]

1. Head and Neck Service, Immunogenomic Oncology Platform, Department of Surgery, Memorial Sloan Kettering Cancer Center, New York, NY, USA
2. Illumina, Inc., San Diego, CA, USA
3. Department of Pathology and Laboratory Medicine, Memorial Sloan Kettering Cancer Center, New York, NY, USA
4. Department of Radiation Oncology, Memorial Sloan Kettering Cancer Center, New York, NY, USA
5. Department of Biostatistics and Epidemiology, Memorial Sloan Kettering Cancer Center, New York, NY, USA
6. Department of Head and Neck Oncology and Surgery, Antoni van Leeuwenhoek Hospital-Netherlands Cancer Institute, Amsterdam, The Netherlands
7. Department of Otorhinolaryngology and Head and Neck Surgery, Leiden University Medical Center, Leiden, The Netherlands
8. Department of Medicine, Memorial Sloan Kettering Cancer Center, New York, NY, USA
9. Department of Oncological Sciences, Icahn School of Medicine at Mount Sinai, New York, NY, USA
10. Lerner Research Institute, Cleveland Clinic, Cleveland, OH, USA

#Co-first authors

Contents:

Supplemental Figures 1–6

Supplemental Tables 1–4

Supplemental Figure 1

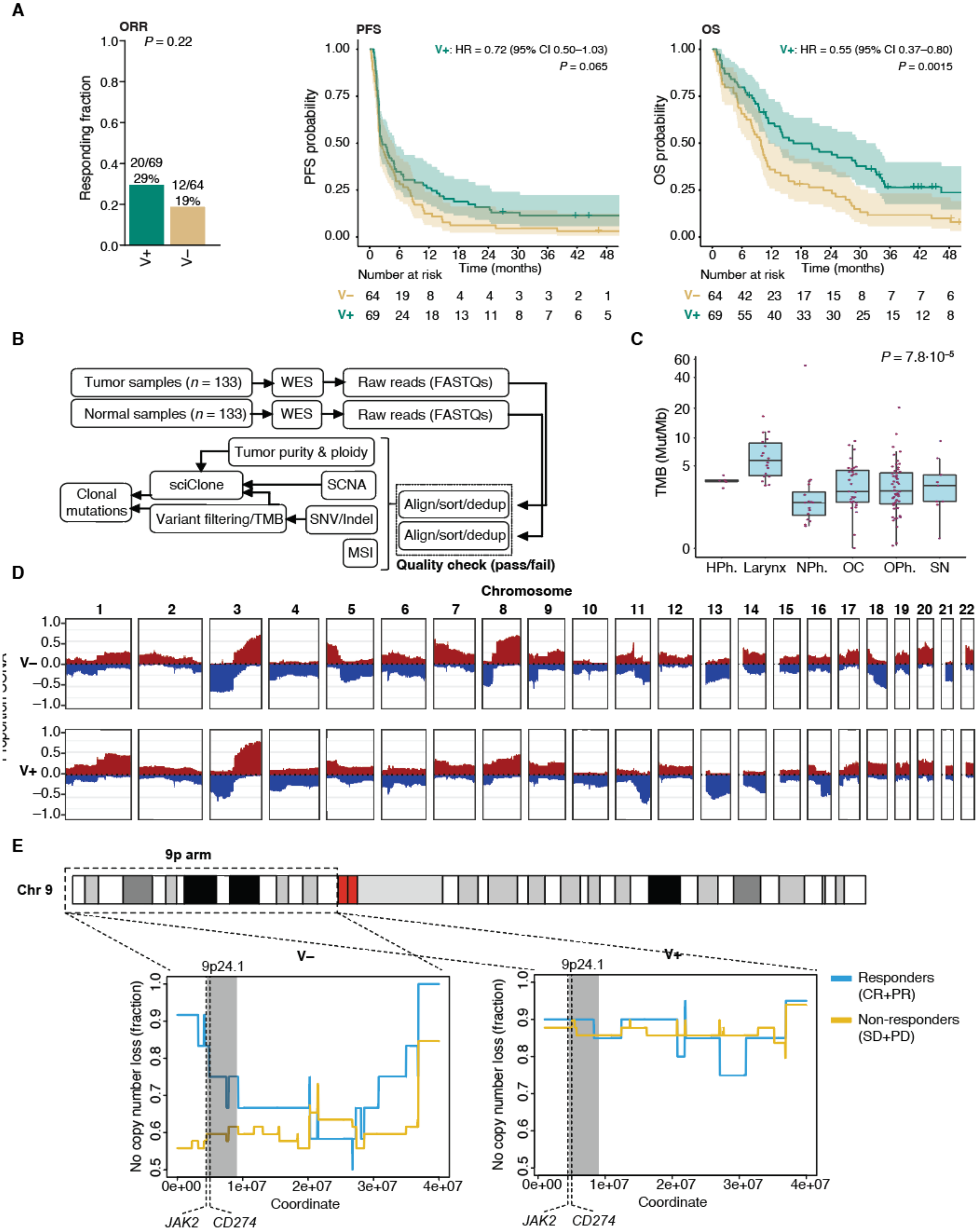

Supplemental Figure 1: Whole-exome sequencing (WES) workflow and additional genomic data for 133 R/M HNSCC main cohort samples

A. ORR, PFS, and OS data for V– tumors (n=64), and HPV+ and EBV+ tumors combined (V+, n=69). The *P*-value for difference in ORR was tested using Fisher's exact test. The hazard ratio and 95% CI for V+ tumors were calculated relative to V– tumors using Cox regression. The *P*-values for PFS and OS were calculated using a logrank test. Shaded represent 95% CIs.

B. The bioinformatics workflow.

C. Boxplots summarizing the total mutational burden (TMB, in muts/Mbp) across different primary HNSCC sites: hypopharynx (HPh), larynx, nasopharynx (NPh), oral cavity (OC), oropharynx (OPh), and sinonasal (SN). Total n=131; tumors located in multiple sites (n=1) or of unknown primary origin (n=1) are not included here. The *P*-value indicates that statistically significant differences in TMB were observed across different sites and was calculated using a Kruskal-Wallis test.

D. Copy number profiles of virus-negative (V–, top row, n=64) and virus-positive (V+, bottom row, n=69) HNSCC samples per chromosome (numbers listed above). The Y-axes represent the frequency of a gain (red; positive values) or a loss (blue; negative values) per locus.

E. Mapping of the 9p arm of chromosome 9 in V– (left; n=64) and V+ samples (right; n=69). Blue and yellow lines demonstrate the fraction of samples without a copy number loss at the locus mapped on the x-axis in responding (complete or partial response) and non-responding patients (stable or progressive disease), respectively. The 9p24.1 locus is highlighted in grey; dotted lines mark the positions of the *JAK2* and *CD274* (encoding the checkpoint PD-L1).

Supplemental Figure 2

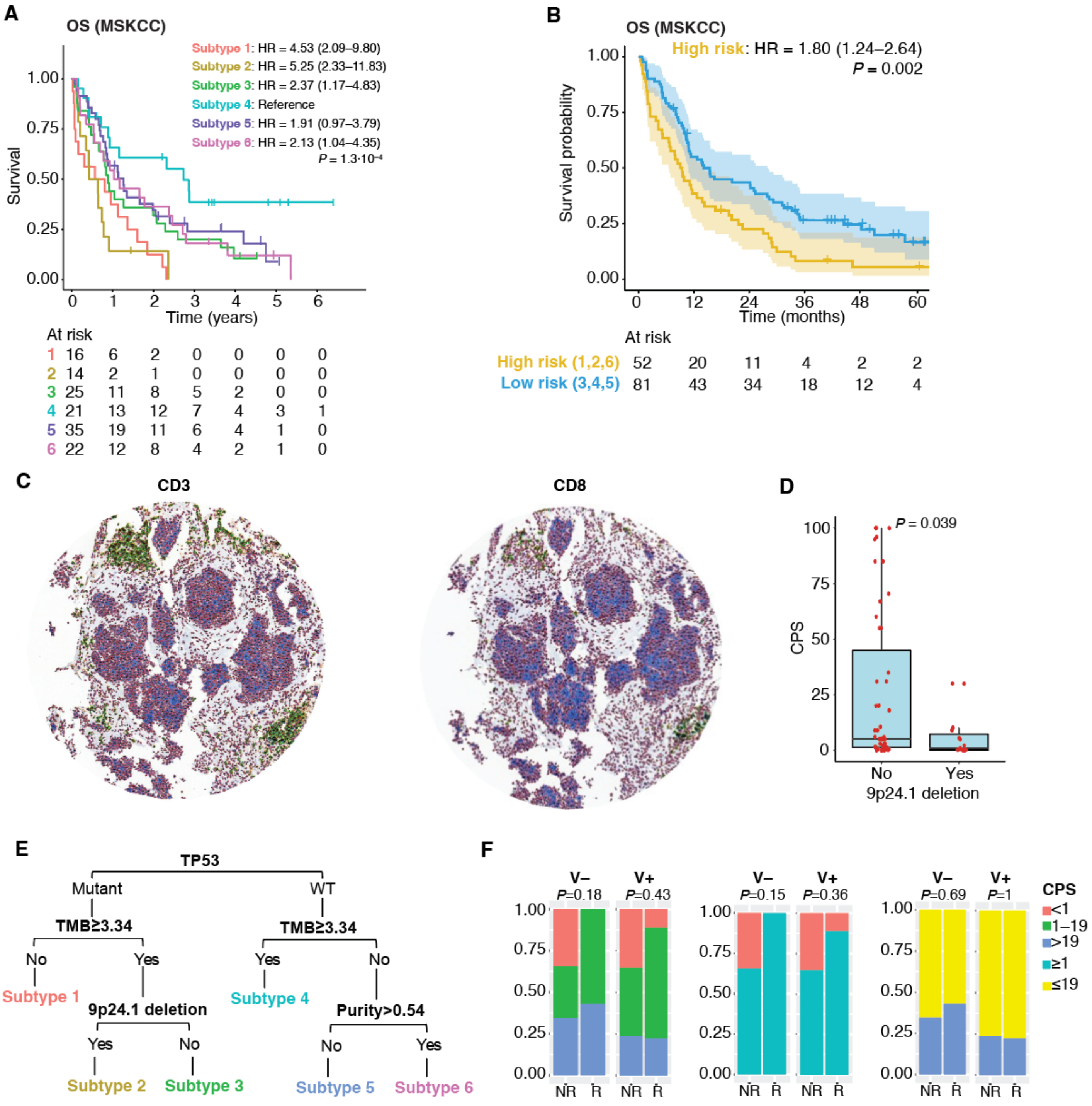

Supplemental Figure 2: Additional data on the molecular subtyping analysis and immunohistochemistry workflow

- **A.** OS estimate per molecular subtype for all 133 R/M HNSCC patients. Hazard ratios and 95% CIs were calculated using Cox regression, using subtype 4 tumors as a reference. The *P*-value indicates that significant differences in OS were observed between the molecular subtypes and was obtained using a logrank test.
- **B.** OS estimates for tumors belonging to subtypes considered high-risk (1, 2, and 6; yellow line) and low-risk (3, 4, and 5; blue line). Hazard ratios and corresponding 95% CIs were calculated using Cox regression, using low-risk tumors as reference. Shaded areas illustrate 95% CIs. *P*-value was obtained using a logrank test.
- **C.** Examples of an intratumoral region of interest (ROI) with a 1mm diameter stained for CD3 (left) or CD8 (right). Positive cells (green) and negative cells (red) are highlighted. Digital image analysis was used to quantify the number of positive cells per ROI automatically.
- **D.** Boxplots illustrating the PD-L1 combined positive score (CPS) values (assessed using immunohistochemistry) for samples (n=133) with and without a 9p24.1 deletion (which includes the *CD274* [PD-L1] gene). The *P*-value was obtained using a Wilcoxon rank sum test.
- **E.** The simplified classifier created to reproduce hierarchical clustering results using recursive partitioning analysis (RPA), which was used for classifying KEYNOTE-012 samples).
- **F.** Stacked bar chart showing the fraction of patients with a relatively high CPS (using various thresholds) among responding and non-responding samples, in the V– (n=64) and V+ groups (n=69). *P*-values were calculated using a Fisher exact test.

Supplemental Figure 3

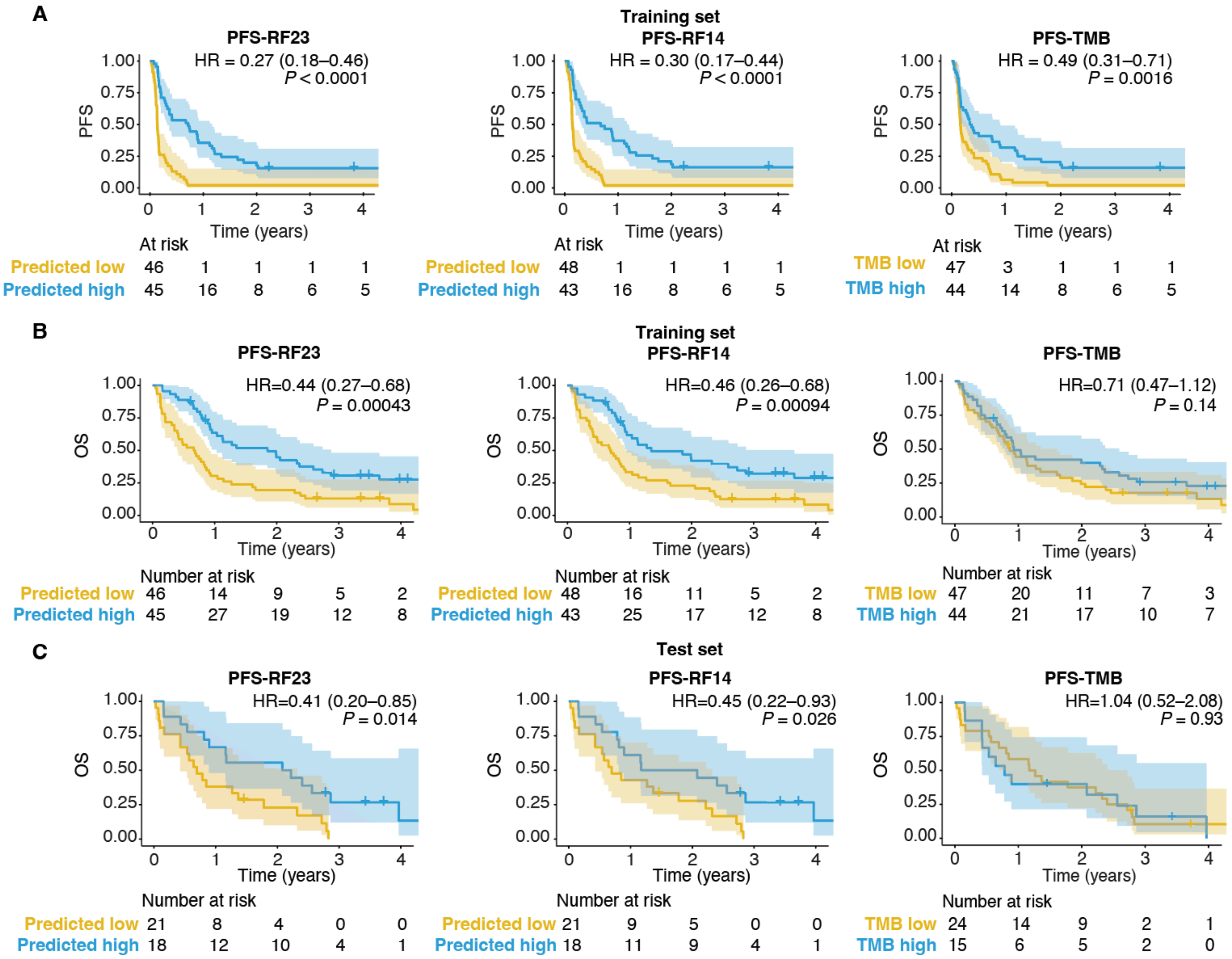

Supplemental Figure 3: PFS data of the PFS-RF23, PFS-RF14, and PFS-TMB models in the training set, and OS data of these models in the training and test set

Hazard ratios and 95% CIs were calculated using Cox regression, using predicted low-survival tumors (yellow) as a reference. *P*-values were obtained using a logrank test. Shaded areas represent 95% CIs.

A. Kaplan-Meier PFS analysis in the training set (n=91) for the PFS-RF23, PFS-RF14, and PFS-TMB models. The median predicted PFS of each model was used as a threshold to divide patients into predicted "high-survival" and "low-survival" groups.

B. Kaplan-Meier OS analysis in the training set (n=91) for the PFS-RF23, PFS-RF14, and PFS-TMB models, separated per the predicted "high-survival" and "low-survival" groups.

C. Kaplan-Meier OS analysis in the test set (n=39) for the PFS-RF23, PFS-RF14, and PFS-TMB models, separated per the predicted "high-survival" and "low-survival" groups.

Supplemental Figure 4

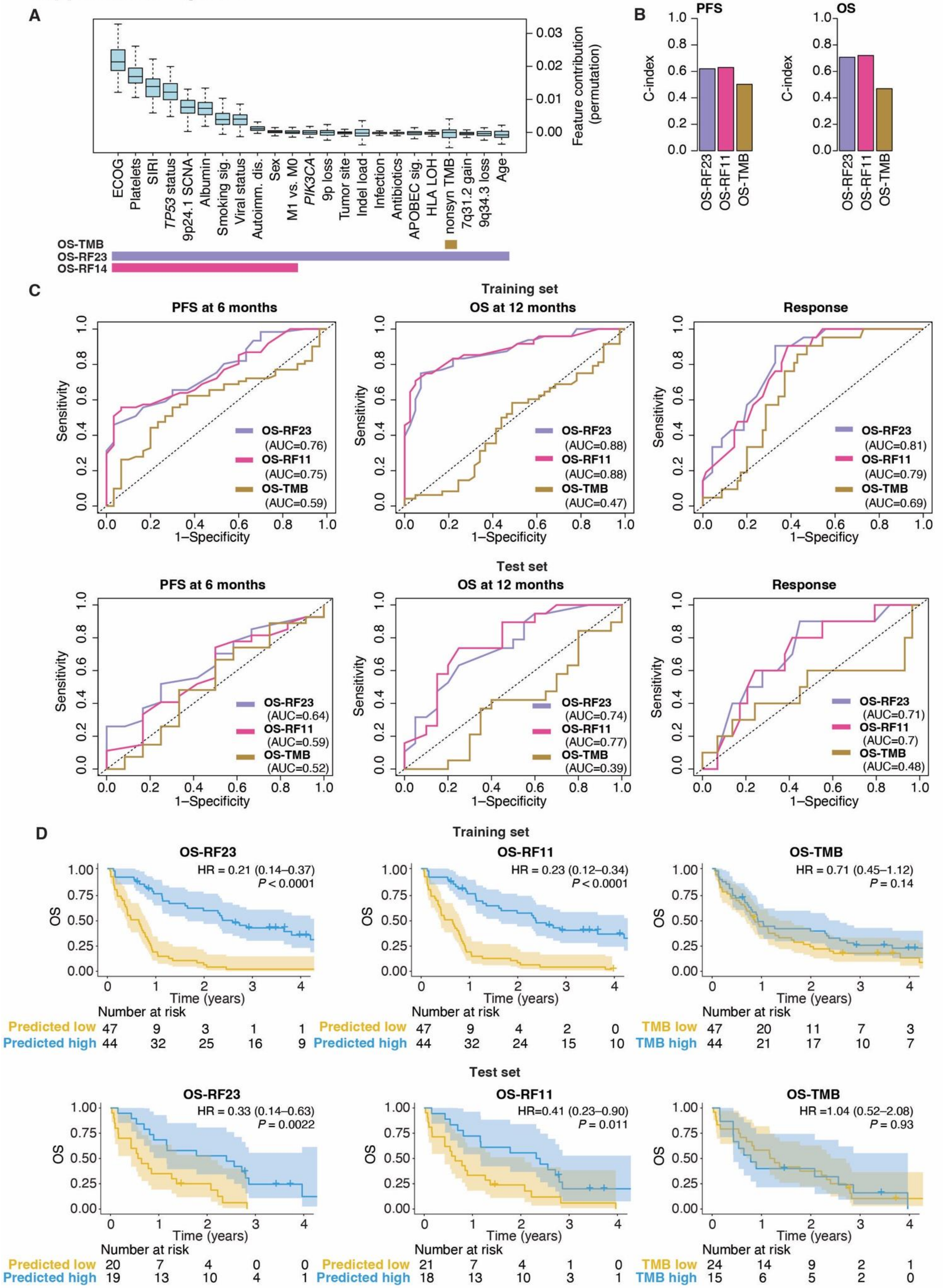

Supplemental Figure 4: Training and testing of an integrated, clinical-genomic model associated with ICB response in HNSCC with OS as outcome

A. Feature contribution of 23 clinical and genomic variables to a random forest classifier predicting OS. Variables are ordered from highest to lowest feature contribution. Colored bars on the left indicate the variables included in the OS-RF23 model (all), the OS-RF11 model (top 11 variables only), and the OS-TMB model (TMB only).

B. Bar charts showing the concordance statistic (C-index) for each model's performance in predicting PFS and OS, calculated in the test set (n = 39).

C. ROC analysis illustrating the performance of the three models (OS-RF23, OS-RF11, and OS-TMB) in predicting 6-month PFS, 12-month OS, and objective response in the 70% training (top row of plots, n=91) and 30% hold-out test set (bottom row, n=39). Three patients were excluded due to incomplete clinical data.

D. Kaplan-Meier OS analysis in the training set (top row, n=91) and test set (bottom row, n=39) for the OS-RF23, OS-RF11, and OS-TMB models. The median predicted OS of each model was used as a threshold to divide patients into predicted "high-survival" (blue line) and "low-survival" groups (yellow line). Hazard ratios and 95% CIs were calculated using Cox regression, using predicted low-survival tumors (yellow) as a reference. *P*-values were obtained using a logrank test. Shaded areas represent 95% CIs.

Supplemental Figure 5

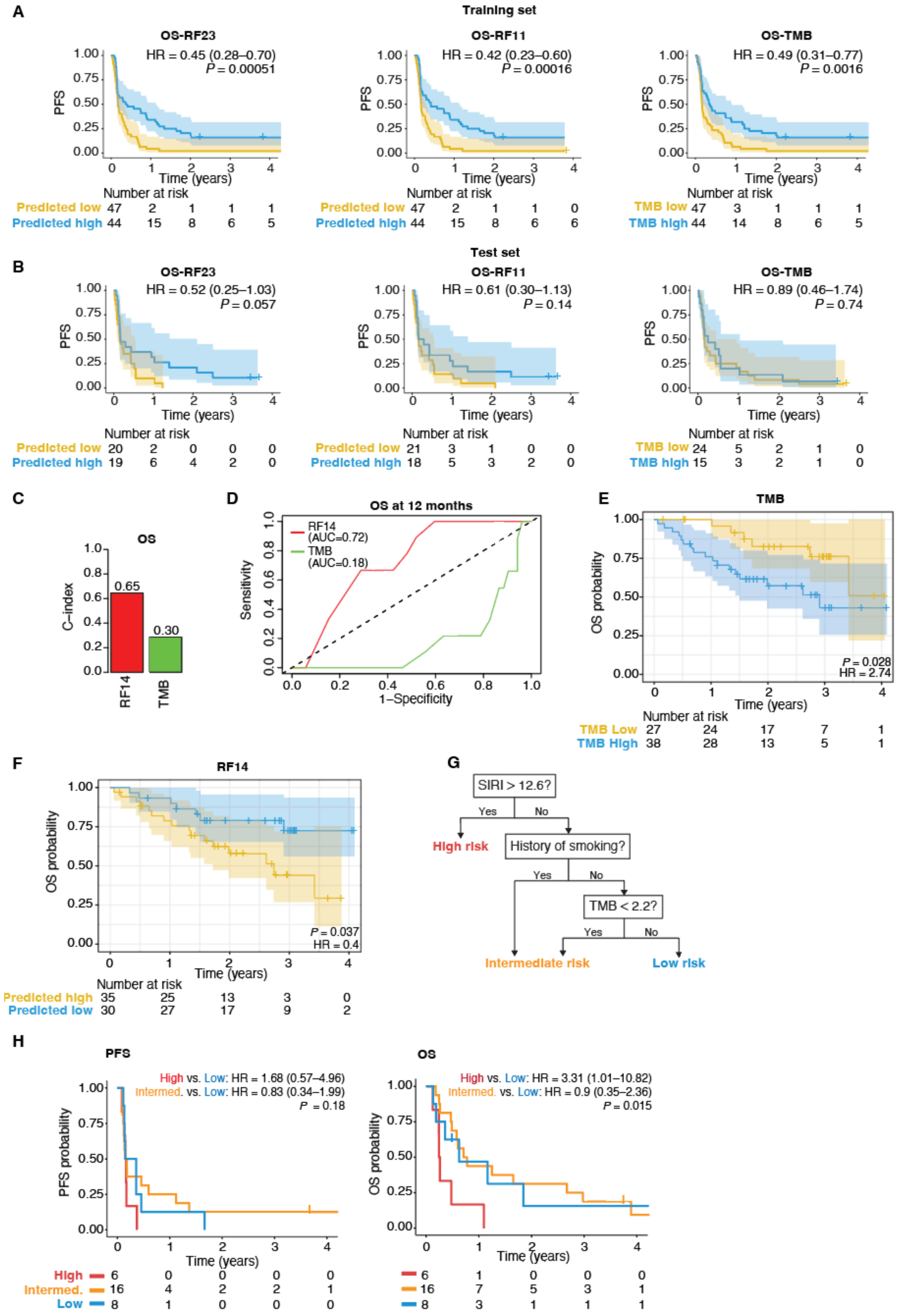

Supplemental Figure 5: The PFS of the OS-RF23, OS-RF11, and OS-TMB models trained on OS in an independent ICB cohort (n=30), OS data of the RF14 and TMB models in an independent non-ICB cohort (n=65), and validation of the RPA classifier in an independent ICB cohort (n=30)

In **A**, **B**, **E**, **F**, and **H**, hazard ratios and 95% CIs were calculated using Cox regression. *P*-values were obtained using a logrank test in these panels, and shaded areas represent 95% CIs.

- **A.** PFS in the training set (top row, n=91) for the OS-RF23, OS-RF11, and OS-TMB models, which were created using OS as outcome. The median predicted OS of each model was used to divide patients into predicted high-survival (blue) and low-survival (yellow) groups.
- **B.** PFS in the test set (n=39) for the OS-RF23, OS-RF11, and OS-TMB models, separated for the predicted high (blue) and low-survival (yellow) groups.
- **C.** C-index for the RF14 and TMB model performance in predicting OS, calculated in the non-immunotherapy cohort.
- **D.** ROC analysis illustrating the performance of the RF14 and TMB models in predicting 12-month OS in the non-immunotherapy cohort (n=65).
- **E.** OS for the TMB model in the non-immunotherapy cohort. The median predicted OS was used to divide patients into predicted “high-survival” (blue) and “low-survival” (yellow) groups.
- **F.** OS for the RF14 model in the non-immunotherapy cohort. The median predicted OS was used to divide patients into predicted “high-survival” (blue) and “low-survival” (yellow) groups.
- **G.** Adaptation of the RPA-based classifier trained in the original cohort (n=131; see **Figure 6E**) using PFS as the dependent variable. The smoking signature was replaced with a smoking history in the independent MSK IMPACT tNGS validation cohort (n=30).

H. PFS (left) and OS (right) of the high, intermediate, and low-risk group obtained using the RPA classifier in the independent validation cohort (n=30).

Supplemental Table 1: Concordance between the molecular subtypes derived from hierarchical clustering (columns) and subtypes predicted by the classifier (rows)

Predicted by classifier (4 features)	Hierarchical clustering (all features, "truth")						
		Subtype 1	Subtype 2	Subtype 3	Subtype 4	Subtype 5	Subtype 6
	Subtype 1	14	2	1	0	0	0
	Subtype 2	0	8	3	0	0	0
	Subtype 3	0	1	18	1	0	1
	Subtype 4	0	3	3	18	2	1
	Subtype 5	2	0	0	2	32	5
	Subtype 6	0	0	0	0	1	15

Supplemental Table 2: Characteristics of the R/M HNSCC patients characterized by MSK-IMPACT tNGS only (n = 30)

Characteristic	No. patients (%)
**Sex**	
Female	5 (16.7)
Male	25 (83.3)
**Age**, median, years (IQR)	63 (53-69)
**Cancer subsite**	
Oral cavity	11 (36.7)
Oropharynx	14 (46.7)
Nasopharynx	1 (3.3)
Larynx	1 (3.3)
Hypopharynx	3 (10.0)
**Smoking history**	
Never	11 (36.7)
Ever	19 (63.3)
**Viral status**	
Negative	16 (53.3)
HPV	13 (43.3)
EBV	1 (3.3)
**Drug class**	
PD-1/PD-L1	29 (96.7)
Combo	1 (3.3)
**ECOG**	
0	13 (43.3)
1	10 (33.3)
2	7 (23.3)
**Stage**	
Non-metastatic	8 (26.7)
Metastatic	22 (73.3)

Supplemental Table 3: Details on the systemic therapies received by the main cohort patients (n = 133) while on ICB therapy

Characteristic	No. patients (%)
**Any systemic treatment while on ICB**	
Yes	19 (14)
No	114 (86)
**Chemotherapy**	
None	123 (92)
Methotrexate	3
5-FU	1
5-FU/Leucovorin	1
Carboplatin/Fluorouracil	1
Carboplatin/Paclitaxel/Cetuximab	1
Fluorouracil/Leucovorin	1
Gemcitabine	1
Paclitaxel	1
**Immunotherapy**	
None	123 (92)
Lirilumab	3
Vopratelimab	2
Ipilimumab/Aldesleukin/Talimogene	1
Ragifilimab	1
MEDI6383 (OX40 agonist)	1
Intralesional aldesleukin	1
EBV-specific CTLs	1

Supplemental Table 4: Univariable analysis with progression-free survival as the dependent variable performed on the 70% training set (n=91) to select features for inclusion in the random forest model

Variable	Training (V+ and V–)<br>*P*-value	Training V+<br>*P*-value	Training V–<br>*P*-value
TP53	0.035	0.397	0.336
PIK3CA	0.045	0.013	0.39
TERT promoter	0.308	0.908	0.954
CD274 SCNA	0.0082	0.2461	0.0179
Del9q34.3	0.106	0.868	0.013
MET amp	0.598	0.446	0.015
Smoking signature (SBS4)	0.001	0.006	0.154
APOBEC signature (SBS2)	0.089	0.07	0.753
TMB	0.14	0.004	0.266
Indel load	0.33	0.23	0.109
Clonal mutational load	0.103	<0.001	0.181
HLA A divergence	0.318	0.69	0.35
HLA B divergence	0.675	0.634	0.943
HLA C divergence	0.874	0.585	0.645
Mean HED	0.577	0.93	0.496
Purity	0.978	0.673	0.821
Ploidy	0.657	0.834	0.599
ITH	0.602	0.866	0.341
Sex	0.48	0.285	0.526
Age at ICB start, in years	0.293	0.995	0.069
ECOG (0 vs. 1–2)	0.02	0.009	0.99
BMI, in kg/m2	0.311	0.497	0.428
Alcohol use	0.493	0.597	0.309
Autoimmune disease	0.123	0.008	0.48

Allergy	0.542	0.084	0.425
Type of tumor (M1 vs. M0)	0.56	0.365	0.446
SIRI, in /nL	<0.001	0.462	<0.001
Platelets, in K/mcL	0.017	0.277	0.044
Hemoglobin, in g/dL	0.866	0.762	0.64
Albumin, in g/dL	0.231	0.862	0.236
Steroids	0.786	0.771	0.509
Infection	0.323	0.148	0.898
Antibiotics	0.442	0.148	0.866
Tumor site (oral cavity vs. others)	0.174	0.022	0.817
HLA LOH	0.246	0.621	0.324
Del9p	0.044	0.384	0.069

Additional genomic features evaluated, where formal hypothesis-testing was not performed due to insufficient statistical power: APM pathway (KEGG 04612) alteration, KEAP1/NRF2 pathway (KEGG 05200) alteration, STK11-AMPK pathway (KEGG 04152+6794) alteration, PTEN pathway (KEGG 04151+6794) alteration, PTPN2-JAK/STAT pathway (KEGG 04630) alteration,

Wnt/beta-catenin pathway (KEGG 04310) alteration, TGF-beta pathway (KEGG 04350) alteration, PD1-PDL1 pathway (KEGG 05235) alteration, *SETDB1* mutation, *SERPINB3* mutation, *SERPINB4* mutation, SBS signature 1 (aging).
